# Neonatal amygdala and fear processing across early childhood

**DOI:** 10.1007/s00787-026-03041-3

**Published:** 2026-04-27

**Authors:** Niloofar Hashempour, Jetro J. Tuulari, Harri Merisaari, John D. Lewis, Saara Nolvi, Tuomo Häikiö, Noora M. Scheinin, Riikka Korja, Hasse Karlsson, Linnea Karlsson, Eeva-Leena Kataja

**Affiliations:** 1 The FinnBrain Birth Cohort Study, Turku Brain and Mind Center, Institute of Clinical Medicine, University of Turku, Turku, Finland; 2 Department of Psychiatry, Turku University Hospital, University of Turku, Turku, Finland; 3 Centre for Population Health Research, University of Turku and Turku University Hospital, Turku, Finland; 4 Department of Psychology and Speech-Language Pathology, University of Turku, Turku, Finland; 5 Department of Psychiatry, University of Oxford, Oxford, UK; 6 Department of Medical Physics, Turku University Hospital, Turku, Finland; 7 Program in Neuroscience and Mental Health, SickKids Research Institute, Toronto, ON Canada; 8 Satakunta Wellbeing Services County, Pori, Finland; 9 Department of Child Psychiatry, Turku University Hospital and University of Turku, Turku, Finland; 10 Department of Public Health, University of Turku, Turku University Hospital, Turku, Finland; 11 The Centre of Excellence for Learning Dynamics and Intervention Research (InterLearn), University of Turku, Turku, Finland

**Keywords:** Amygdala, Eyetracking, MRI, Emotional processing, Fear processing

## Abstract

The amygdala plays a crucial role in emotional processing, particularly in detecting threat-related stimuli and regulating responses to them. Fear processing is a vital function emerging during the latter half of the first postnatal year and becomes progressively more regulated and context-dependent with maturation across early childhood. The neural underpinnings of early-emerging individual differences in fear processing remain underexplored. Our previous study showed an association between newborn left amygdala volume and increased disengagement from fearful vs. non-fearful faces at 8 months. This study builds on our previous findings by extending the analysis longitudinally. We investigated whether neonatal amygdala volume and microstructural properties, indexed by mean diffusivity, are associated with attentional biases toward fearful faces at 30 and 60 months. Neonatal MRI was acquired at 2–8 weeks of age using 3T MRI. The same cohort completed eye-tracking at follow-ups (*n* = 57 at 30 months; *n* = 54 at 60 months). Our results show that larger newborn left amygdala volume was associated with decreased disengagement from fearful (vs. non-fearful) faces at 30 months (*p* = .041), but not at 60 months (*p* = .553). Moreover, sex-specific analyses indicated that higher mean diffusivity in the left amygdala was associated with lower fear bias at 60 months in boys (*p* = .046). These findings highlight the dynamic nature of amygdala-related fear processing across early development. Associations between neonatal amygdala characteristics and fear bias appeared age-dependent and sex-specific, consistent with developmental changes in fear processing, with fear bias typically elevated in infancy and becoming less pronounced by around five years of age.

**Supplementary Information:**

The online version contains supplementary material available at https://doi.org/10.1007/s00787-026-03041-3.

## Introduction

Both adult humans and primates respond more rapidly to threat-related stimuli, such as snakes, spiders, and angry faces, compared to neutral stimuli [1]. This heightened sensitivity supports the hypothesis proposed by Öhman and Mineka [2] regarding an inborn fear module, suggesting that humans are biologically predisposed to process threatening stimuli rapidly and without conscious awareness. Furthermore, this predisposition is normative and emerges early in development [3, 4]. Infants show differential responsiveness to evolutionarily relevant stimuli like angry voices or hissing snakes [5] and can categorize basic emotional expressions [6, 7]. By around 6 months of age, infants begin to focus more on threat-related cues, particularly fearful faces [8–10]. This perceptual bias aids vigilance as infants explore beyond their safe spaces, with the onset of locomotion marking their transition to autonomy and risk assessment [11]. However, while this heightened sensitivity is evident early in development, it is not static. Over time, fear-related attention biases decline across the first five years of life [12, 13], although traces of these biases may persist into later developmental stages in some individuals and may have implications for socioemotional development and anxiety-related disorders [14, 15]. To assess these developmental changes in attention to emotional cues, eye-tracking offers a noninvasive and developmentally appropriate method for measuring how infants and young children allocate attention to different facial expressions [16]. Metrics such as looking time, dwell proportion, and disengagement latency capture both rapid orienting and sustained attention to emotionally salient stimuli [16]. Eye-tracking is used to quantify fear processing, and it is equally suitable for measuring attention to a range of emotional expressions, including angry, happy, and neutral faces [17]. Fearful and angry faces tend to elicit particularly strong attentional responses due to their evolutionary relevance [18]. Yet little is known about how attention to different emotional expressions develops beyond infancy as longitudinal evidence remains limited.

The amygdala is well known for its role in recognizing and processing fear, as well as in the learning and expression of conditioned fear [19]. While the ability to detect facial expressions emerges early in infancy [20], the mechanisms underlying developmental changes in this ability remain an active area of research [21–25]. To date, only two studies, both from our group, have been published on the role of amygdala volume and diffusion characteristics in the variation of fear processing during early childhood [26, 27]. These studies demonstrated that neonatal amygdala properties were associated with individual differences in fear-related attention at 8 months, a period when fear responses intensify in normative development [28].

The functional lateralization of the amygdala has been well documented in both adult and developmental neuroimaging research [29, 30]. The left amygdala has been associated with more sustained, detailed, and cognitively modulated processing of emotional stimuli, whereas the right amygdala is thought to respond more rapidly and automatically to threat-related cues [31, 32]. This pattern has been interpreted to reflect the right amygdala’s role in automatic detection of affective salience and the left amygdala’s involvement in elaborative evaluation of emotional meaning. Developmentally, these lateralized functions may emerge early, as the infant brain increasingly integrates perceptual and regulatory systems [33]. Early structural variation in the left amygdala may relate to individual differences in attentional engagement and disengagement to emotional cues [26, 34], while with increasing age, right-lateralized processes involved in rapid perceptual detection may become more prominent [35]. Thus, early differences in left and right amygdala structure and microstructure could have distinct implications for the trajectory of emotional reactivity and regulation across childhood.

In the present study, we extended our previous work by taking a longitudinal approach to investigate emotional face processing in children using the emotional overlap paradigm [36]. Specifically, we examined whether neonatal structural and diffusion MRI metrics of the amygdala measured at 2–8 weeks of age predict fear processing later in development. Fear processing was assessed via eye-tracking at 30 and 60 months. We hypothesized that larger left amygdala volume would predict lower disengagement from fearful faces at 30 months, reflecting sustained attention to threat cues, and that this association would no longer be evident at 60 months due to developmental changes (e.g., a clear reduction in fear bias [13]), in fear-related attention across early childhood. Finally, we explored potential sex differences in these associations; these analyses are considered exploratory but are motivated by prior evidence of sex-specific trajectories in emotion regulation during early childhood [27, 37, 38].

## Methods

This study is part of the ongoing FinnBrain Birth Cohort Study (www.finnbrain.fi), which aims to examine the effects of early life stress, such as prenatal distress, on children’s brain development and long-term health [39]. The study was conducted in accordance with the Declaration of Helsinki and received approval from the Ethics Committee of the Hospital District of Southwest Finland (MRI: § 95, ETMK:31/180/2011; Eye tracking: § 322, ETMK:107/180/2012). Informed consent was obtained from the mothers on behalf of their children.

### Participants

Pregnant women were recruited during their first ultrasound visit around the 12th week of gestation at three maternal welfare clinics offering ultrasound services for expectant mothers delivering at Turku University Hospital, as well as in the Åland Islands, Finland, between December 2011 and April 2015 [39]. The inclusion criteria for the study were as follows: (a) an ultrasound-verified pregnancy, and (b) sufficient proficiency in Finnish or Swedish. A total of 189 infants underwent MRI scans between 2 and 8 weeks after birth. Eligible participants had a birth weight ≥ 2500 g and a gestational age of at least 36 weeks. Infants with known congenital central nervous system abnormalities or abnormal findings on earlier MRI examinations were excluded. Of the initial cohort, 64 were removed due to motion-related artifacts [40]. A total of 908 families were invited to participate in the eye-tracking study. Of these, 694 families (76.4%) were successfully contacted, and 488 (70.3%) agreed to participate. Ultimately, 437 families (63.0% of those reached; 89.5% of those who had agreed) completed the laboratory visit, during which 421 eye-tracking sessions were conducted. Of the 421 infants, 31 (7.4%) did not yield usable data due to fussiness or technical issues. Among the remaining participants, 363 infants (93.1%; 46.0% girls) contributed at least three valid trials for each stimulus condition and were included in the final analytic sample. Only children who had undergone MRI scans at 2–8 weeks of age, in addition to a valid eye-tracking assessment at either 30 or 60 months, were included in the current analysis [41]. The study sample included overlapping participants with both MRI and eye-tracking data available for 57 children at 30 months and 54 children at 60 months. Within this group, diffusion weighted imaging (DWI) and eye-tracking data were available for 42 children at 30 months and 37 children at 60 months. Participants excluded from the final analytic sample due to unusable MRI data (e.g., motion-related artifacts) or insufficient eye-tracking quality did not differ from those included with respect to available baseline characteristics, including maternal age, education, prenatal substance use, gestational age at birth, or infant sex. All children included in the present analyses had participated in both MRI and eye-tracking assessments. Participant characteristics are presented in Table 1.

**Table 1:** Participant characteristics. Values are mean (SD) unless otherwise indicated. Ranges (min–max) were as follows: age at MRI 2.00–7.71 weeks; birth weight 2530–4700 g (30 months) and 2580–4670 g (60 months); intracranial volume 517,422–715,641 mm³ (30 months) and 517,422–715,641 mm³ (60 months)

Characteristic	30 months	60 months
Child characteristics		
Sex, n (%) boys	32 (61.5%)	26 (54.2%)
Age at MRI (weeks)	3.66 (1.08)	3.58 (1.16)
Birth weight (g)	3464 (478)	3476 (428)
Intracranial volume (mm³)	619,485 (45,567)	617,139 (46,762)
Maternal characteristics		
Maternal age at delivery (years)	30.65 (4.58)	29.75 (4.53)
Maternal education, n (%)		
Low–mid (levels 1–5)	9 (17.3%)	11 (20.4%)
High/vocational (level 6)	21 (40.4%)	20 (37.0%)
High (levels 7–9)	22 (42.3%)	23 (42.6%)
Tobacco use during pregnancy	0 (0%)	0 (0%)
Alcohol use during pregnancy	5 (9.8%)	7 (13.0%)
Drug use during pregnancy	0 (0%)	0 (0%)
SSRI/SNRI use during pregnancy	3 (6.0%)	3 (5.8%)

### Magnetic resonance imaging (MRI)

MRI scans were performed on newborns between 2 and 8 weeks of age (*Mean = 3.65 weeks*,* SD = 1.06)* at the Medical Imaging Center of the Hospital District of Southwest Finland, from November 2012 to January 2016. Imaging was conducted using a Siemens Magnetom Verio 3 T scanner. Structural imaging included axial proton density (PD)-T2 turbo spin echo (TSE) and sagittal 3D T1 magnetization-prepared rapid acquisition gradient echo (MPRAGE) sequences, both with isotropic 1.0 mm³ resolution. The PD-T2-TSE sequence utilized echo times (TE) of 13 ms and 102 ms, while the 3D T1 MPRAGE sequence had a repetition time (TR) of 1,900 ms and a TE of 3.26 ms. Both sequences employed the “whisper” gradient mode to reduce noise and enhance comfort for the participants [42, 43].

### Diffusion weighted imaging (DWI)

DWI was conducted with 96 diffusion directions, split into three segments. A selection of 60 directions at b = 1000 s/mm², along with three b = 0 s/mm² images, provided robust tensor fitting for infant data. Each of the three segments included a b0 image at the beginning, middle, and end, resulting in a total of 9 b0 images across the entire protocol. The sequences used a 2 mm³ isotropic resolution, and motion and eddy current corrections were performed with FSL tools. Motion effects were addressed through preprocessing and subsequent quality-control procedures, including automated correction, visual inspection, and exclusion of scans that did not meet predefined quality criteria. In addition, the 96-direction diffusion acquisition was divided into three segments. This segmentation was implemented to allow the scan operator to monitor participant motion during data acquisition and, if necessary, repeat a segment in which excessive motion occurred. Using shorter acquisition segments made it feasible to repeat affected portions of the scan when needed, whereas repeating a single continuous 96-direction sequence would have been impractical. After acquisition, the three acceptable segments were combined to obtain the full 96-direction dataset [43]. Although fractional anisotropy (FA) and axial diffusivity (AD) are widely used DTI metrics, their interpretation in gray-matter regions such as the amygdala is limited [44, 45]. Given the isotropic diffusion characteristics of gray matter, FA and AD should be interpreted with caution, whereas MD provides a more reliable index of microstructural properties in the amygdala and other gray-matter structures [42, 45, 46]. Therefore, MD metrics were derived from the diffusion data, which were aligned with the T1-weighted images to extract amygdala-specific measures. The amygdala labels, originally defined on T1- and T2-weighted images of an infant brain atlas, were non-linearly registered to each subject’s native T1-weighted image to enable accurate region-specific extraction. Erosion was applied to the amygdala labels to minimize partial volume effects, allowing for accurate DTI analysis [42].

### Template creation

A population-specific template was developed using data from 125 MRIs, following methods similar to previous methods [47]. This involved iterative construction with T1 scans, followed by registration and averaging of T2 scans. The template was manually labeled for all subcortical structures [40], and accuracy was confirmed through the generalized conformity index. Subject-specific labeling followed the label fusion technique [48], with adjustments for anatomical heterogeneity across hemispheres. The final segmentations were obtained using patch-based label fusion [49, 50], ensuring precise identification of the amygdala in individual subjects [51].

### Eye-tracking assessments at 30 and 60 months

Eye-tracking assessments were conducted in a dimly lit room using a 19” CRT monitor and an EyeLink1000 + eye tracker (SR Research Ltd, Toronto, Ontario, Canada) with a sampling frequency of 500 Hz. At 30 months, the child sat on the caregiver’s lap, and at 60 months, the child sat alone on a chair, positioned 50–70 cm from the eye tracker. The researcher, seated at a separate host computer behind a curtain to prevent interference, initiated the trial manually after the child’s gaze was centered on the screen.

Prior to measurement, a five-point calibration procedure was performed using an audiovisual animation presented sequentially at five locations on the screen. Calibration was repeated if the tracker failed to detect the eye due to excessive movement, and again during the measurement, if needed. Short breaks were provided if necessary.

**Fig. 1:**
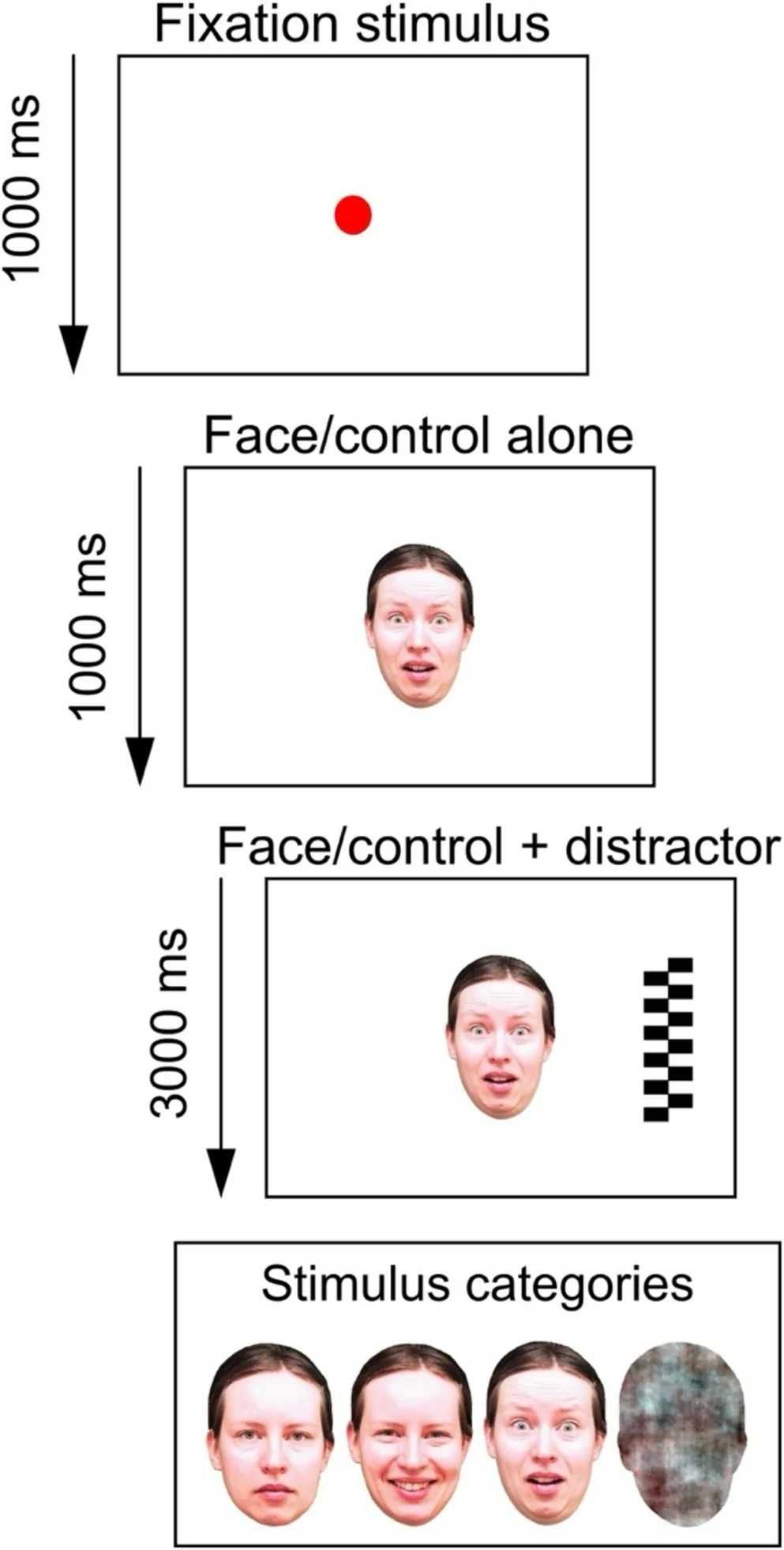
Illustration of the eye-tracking paradigm used to assess infants’ attention to social emotional signals. After the infant fixated on a central stimulus (red circle), either a face or a scrambled face pattern was presented, followed by a high-contrast lateral distractor. The probability of attentional disengagement from the central stimulus to the lateral stimulus was derived from the eye-tracking data and used as an index of attention to scrambled face patterns as well as to neutral, happy, and fearful faces

**Fig. 2:**
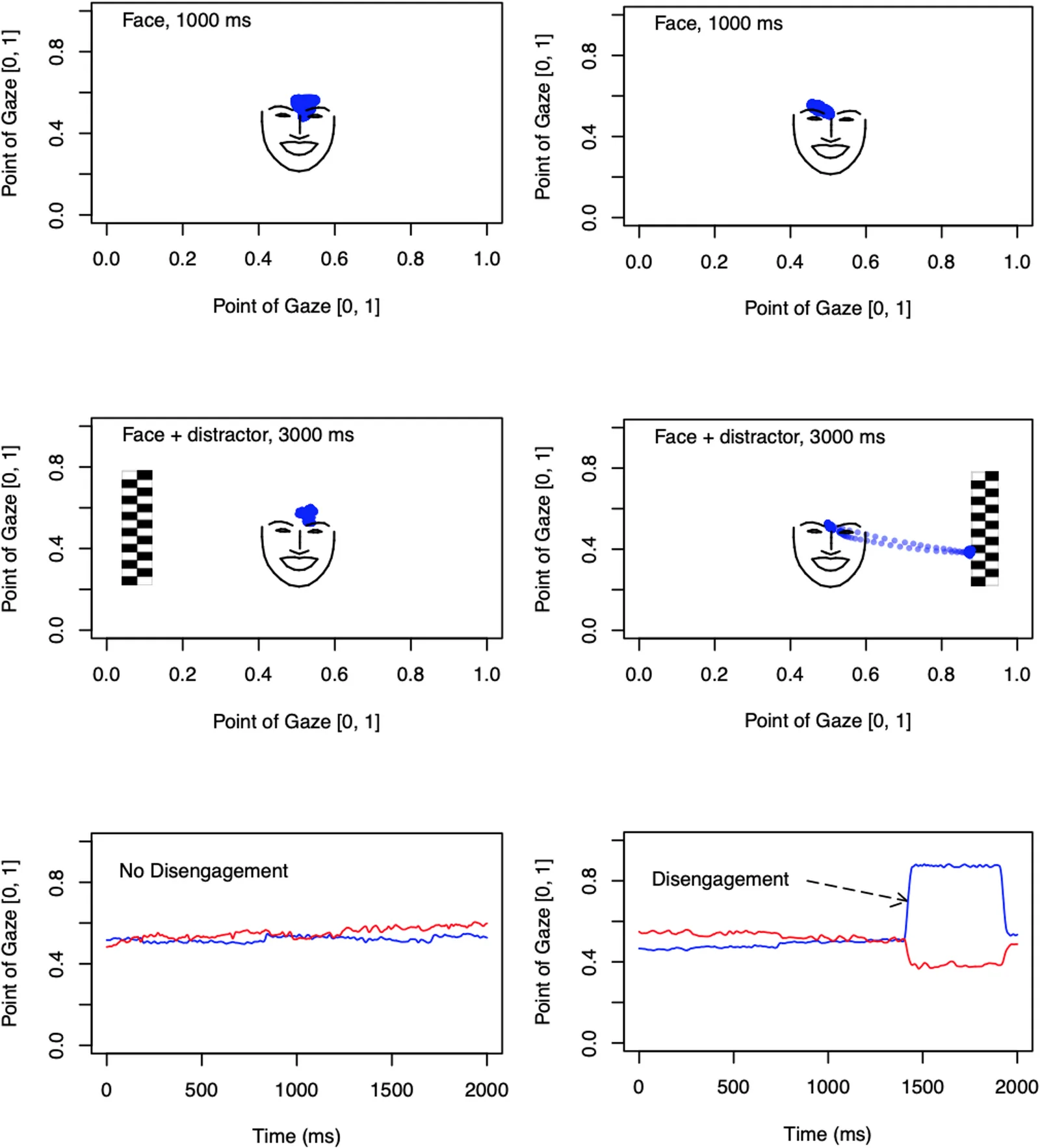
Illustration of the overlap paradigm and data from two example trials. Top panel: Participants were presented with a face or a non-face in the center of the screen. Middle panel: One second later, a “distractor” was added to the left or to the right and shown for an additional 3000 ms. Lower panel: example data from two trials. The time-series plots (0–2000 ms) illustrate gaze behavior during the face-only period (0–1000 ms) and the first 1000 ms following distractor onset, which was used to determine disengagement. Left: An example of a “no disengagement” trial in which the gaze does not shift from the central to the lateral stimulus. Right: An example of a “disengagement” trial in which the gaze shifts rapidly from the central to the lateral stimulus. The X- and Y-coordinates of the point of gaze on the display are shown by the blue and red lines, respectively. Point of Gaze is measured as proportion of the whole viewing area so that values below 0.5 denote looking left or below of the center (blue and red lines, respectively) with values above 0.5 denote looking right or above of the center (blue and red lines, respectively)

The overlap paradigm [52] assessed the child’s attention disengagement from faces and non-faces to distractors (Figs. 1 and 2). In each trial, a neutral, happy, or fearful face, or a non-face control stimulus, was displayed centrally for 1000 ms, followed by a salient lateral distractor (a black-and-white checkerboard pattern or empty and filled circles) appearing on either the left or right side of the screen for 3000 ms. Both types of lateral distractors were used at 30 and 60 months and were selected randomly for each trial. To enhance attentional capture, animated distractors were used, with slight variations in animation across measurement points. One trial lasted 4000 ms, with the sizes of the central and lateral stimuli being 15.4° x 10.8° and 15.4° x 4.3°, respectively. A brief animation was shown before each trial to capture the child’s attention, and the trial was initiated once the child’s gaze was centered on the screen.

The lateral distractor stimulus presentation differed between visits: at 30 months, the distractor’s contrast polarity was reversed at a frequency of 10 Hz to increase saliency, and this flashing took place for the whole duration of distractor being presented. While at 60 months, the distractor was otherwise the same, it only began flashing after the child directed attention towards it [12, 53]. The central stimuli were semi-randomized, with the constraint that no stimulus was presented more than three times in a row, and the lateral distractor was selected randomly for each trial. Six trials per condition were presented, totaling 24 trials.

### Preprocessing of eye-tracking data

The trial data, consisting of timestamps for the onset of central and lateral images and the xy coordinates of participants’ gaze positions (500 samples per second), were stored as text files and analyzed using a MATLAB library (MathWorks, Natick, MA) [54]. The following criteria were applied to ensure the quality of the trials retained for analysis. First, trials were required to show sufficient attention to the central stimulus (> 70%) during the time interval starting at the onset of the trial (i.e., appearance of the face or non-face) and extending until the end of the analysis period, defined as the gaze disengagement from the central to the lateral stimulus, or if no disengagement was observed, 1000 ms after the appearance of the lateral distractor. The 1000 ms analysis window was defined a priori based on previous overlap-paradigm studies using similar methodology [41, 55]. This time frame was chosen to capture rapid, stimulus-driven attentional disengagement while minimizing the influence of later voluntary or strategic gaze shifts. The remaining 2000 ms of stimulus presentation ensured consistent trial duration but were not included in the primary disengagement measure. Second, trials needed to include enough valid gaze samples with no gaps exceeding 200 ms. Gaps smaller than 200 ms were extrapolated by the analysis script using the last recorded sample, but gaps greater than 200 ms flagged the trial as invalid, excluding it from subsequent analyses. Third, for trials to be included, the exact timing of the eye movement from the central to the lateral stimulus had to be identifiable. If the eye movement occurred during a period of missing or extrapolated gaze data, the trial was rejected. These parameters were established a priori based on prior studies using the same methodology and analytic approach [54, 56].

### Eye-tracking measures

*Disengagement Probabilities (DPs):* Eye-tracking data were first coded into a binary disengagement value (0/1) based on whether the gaze shifted from the central to the lateral stimulus (i.e., disengagement probability, DP). The trial-level data was then aggregated to estimate the mean disengagement probability across different experimental conditions. Mean DPs in the scrambled-control picture, happy, neutral, and fearful face conditions were used as primary measures of disengagement.

*Fear Bias Score: *A Fear Bias Score was calculated as the mean difference in DPs between the happy and neutral face (i.e., non-fearful face) conditions compared to the fearful face condition. Higher Fear Bias scores indicate greater difficulty disengaging from fearful faces relative to happy and neutral faces. This approach has been used in previous infant studies where the DP levels are typically equal for neutral and happy faces and lowest for fearful faces [13]*.*

*Emotion-Specific Bias Scores: *To better align with age-typical patterns of DPs, additional bias scores were calculated [13]. These scores were derived by subtracting the DPs for each facial expression condition from the DPs for the scrambled-control picture: NE_bias (neutral face), HA_bias (happy face), and FE_bias (fearful face). Higher values reflect increased difficulty disengaging attention from emotional faces relative to the control stimulus.

### Statistical analyses

All statistical analyses were conducted using IBM SPSS Statistics version 26 (IBM Corp., Armonk, NY). Parametric methods were employed to examine the associations, as the distribution of the primary variables of interest did not show significant deviations from normality. Normality of the data was assessed using histogram inspection, the Kolmogorov–Smirnov test, and evaluation of skewness and kurtosis, which indicated that the data were normally distributed. Although disengagement probabilities (DPs) are bounded between 0 and 1, they represent child-level means across trials and showed no substantial skewness or ceiling/floor effects. Accordingly, linear regression models were used to examine associations of bilateral amygdala volume and mean diffusivity (MD) with:

1. DPs in different experimental conditions (control, neutral, happy, and fearful face conditions).
2. Fear bias scores (difference in DPs between happy/neutral vs. fearful face conditions).
3. Emotion-specific bias scores (neutral bias, happy bias, and fearful bias, calculated by subtracting DPs in each condition from the scrambled-control picture).

The analyses included gestational age at the time of the MRI scan to assess its impact. Infant sex was also tested as an interaction term with amygdala volume and MD to explore potential sex-specific effects within the statistical models. Given the exploratory nature of this analysis, no correction for multiple comparisons was applied [57]. For all statistical analyses, 95% confidence intervals were calculated to estimate the precision of the effect sizes.

## Results

### Descriptive characteristics

Amygdala volumes were correlated with age at MRI (left amygdala: *r* =.325, *p* =.013, right amygdala: *r* =.273, *p* =.040). The correlation between left and right amygdala volumes was high (*r* =.739, *p* <.001). MD of the right and left amygdalae were also strongly correlated (*r* =.653, *p* <.001). MD in the left amygdala, but not the right, was negatively associated with gestational age at MRI (*r* = −.470, *p* =.002). Notably, no significant correlations were found between amygdala volumes and amygdala MD. Descriptive statistics for amygdala volumes, MD, and eye-tracking measures are provided in Tables 2 and 3.

**Table 2:** Eye-tracking descriptive statistics

30 months 60 months								
Variable	Mean	Min	Max	Std	Mean	Min	Max	Std
DP, happy face	0.83	0.40	1.00	0.17	0.70	0.17	1.00	0.22
DP, fearful face	0.70	0.00	1.00	0.26	0.59	0.00	1.00	0.25
DP, neutral face	0.74	0.00	1.00	0.22	0.69	0.00	1.00	0.24
DP, control non-face	0.84	0.21	1.00	0.20	0.81	0.33	1.00	0.20
HA_bias	−0.01	−0.60	0.67	0.24	0.10	−0.50	0.50	0.24
FE_bias	−0.14	−1.00	0.33	0.27	0.21	−0.40	1.00	0.27
NE_bias	−0.10	−1.00	0.50	0.26	0.11	−0.40	0.50	0.22
Fear bias	0.08	−0.63	0.67	0.23	0.10	−0.58	0.71	0.24

*DP* Disengagement probability, Fear bias = Bias for fearful vs. non-fearful (happy+neutral), NE_bias = Bias for neutral vs. control face; HA_bias = Bias for happy vs. control face picture; FE_bias =Bias for fearful vs. control face, *Std* Standard deviation.

**Table 3:** MRI descriptive statistics

MRI at 2–8 weeks of age				
Variable	Mean	Min	Max	Std
Left amygdala volume	264.58	188.00	344.81	37.30
Right amygdala volume	261.65	181.89	344.60	36.40
Left amygdala Mean diffusivity (×10⁻³ mm²/s)	1.08	1.00	1.26	0.044
Right amygdala Mean diffusivity (×10⁻³ mm²/s)	1.09	1.01	1.23	0.042

### The associations between the left and right amygdala volumes and attention disengagement from faces and fear bias at 30 and 60 months

**Fig. 3:**
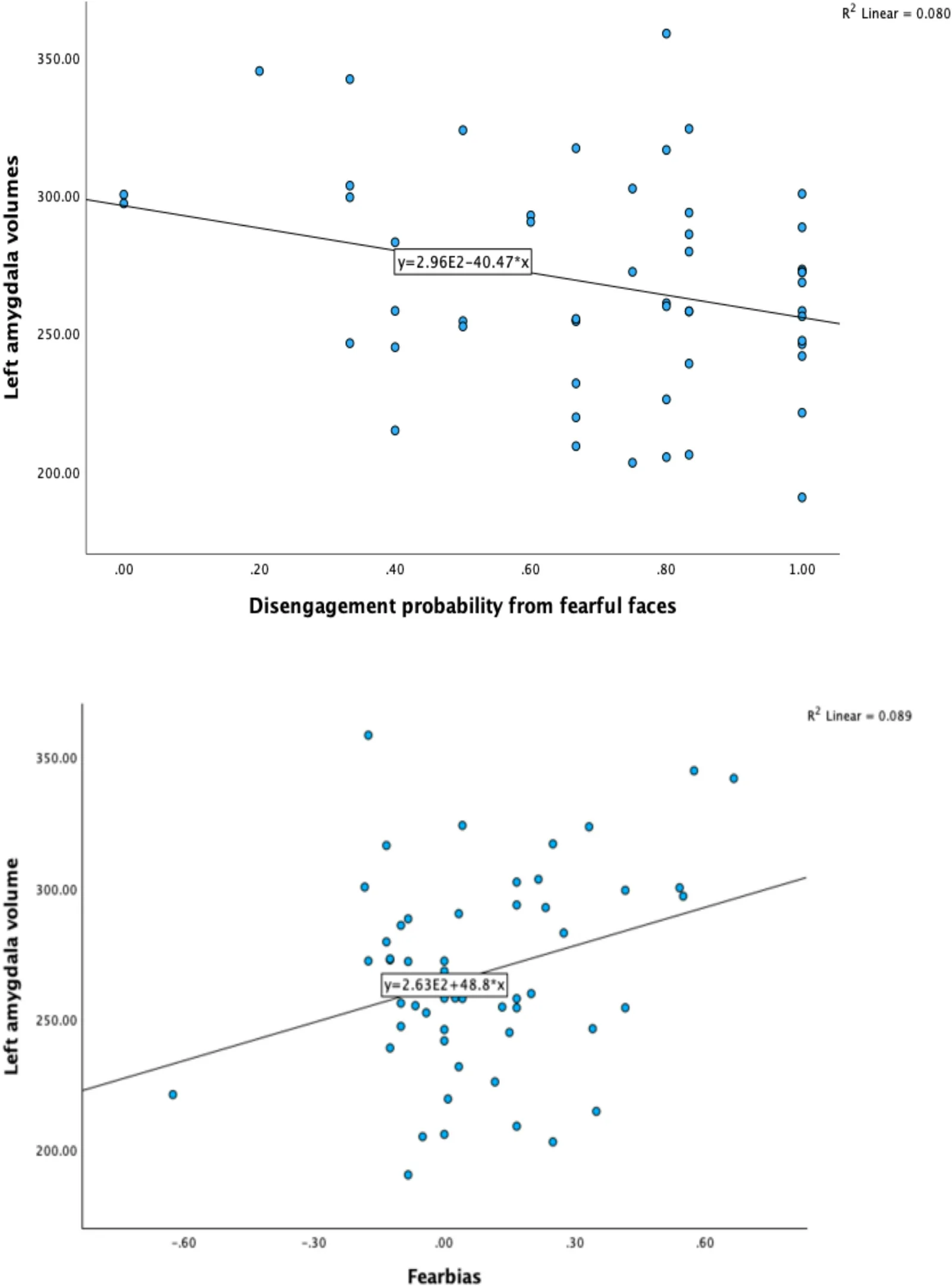
Associations between the left amygdala volume and disengagement probability from fearful faces and fear bias at 30 months

At 30 months, the left amygdala volume adjusted for ICV correlated with DPs in fearful face condition when age at scan time was controlled for (β = −0.058, *p* =.041, SE = 0.027, 95% CI [–0.114, –0.003]; model: F(2, 29) = 3.44, *p* =.046, R² = 0.192), indicating that DPs in the fearful face condition and age at scan explained approximately 19.2% of the variance in left amygdala volume (Fig. 3) (supplementary Table 1). The regression equation was: Left amygdala = 0.502 − 0.058 (Disengagement probability from fearful faces) − 0.009 (age at scan).

Moreover, the left amygdala volume adjusted for ICV correlated significantly with fear bias at 30 months after age at scan was controlled for (*β = 0.073, p =.033, SE = 0.033, 95% CI [0.006, 0.141]; model: F(2, 29) = 3.67, p =.038, R² = 0.202)*, indicating that fear bias and age at scan explained approximately 20.2% of the variance in left amygdala volume (Fig. 3) (supplementary Table 1). The regression equation was: Left amygdala = 0.455 + 0.073(Fear bias) − 0.009 (age at scan).

The right amygdala volume adjusted for ICV did not show a significant correlation with DPs in any condition or with fear bias (supplementary Table 2). At 60 months, neither the left nor right amygdala volumes were associated with DPs or fear bias. We did not observe any significant interaction effects with sex at both time points. All analyses, both significant and non-significant, are presented in the supplementary tables.

### The associations between the left and right amygdala volumes and emotion-specific bias scores

**Fig. 4:**
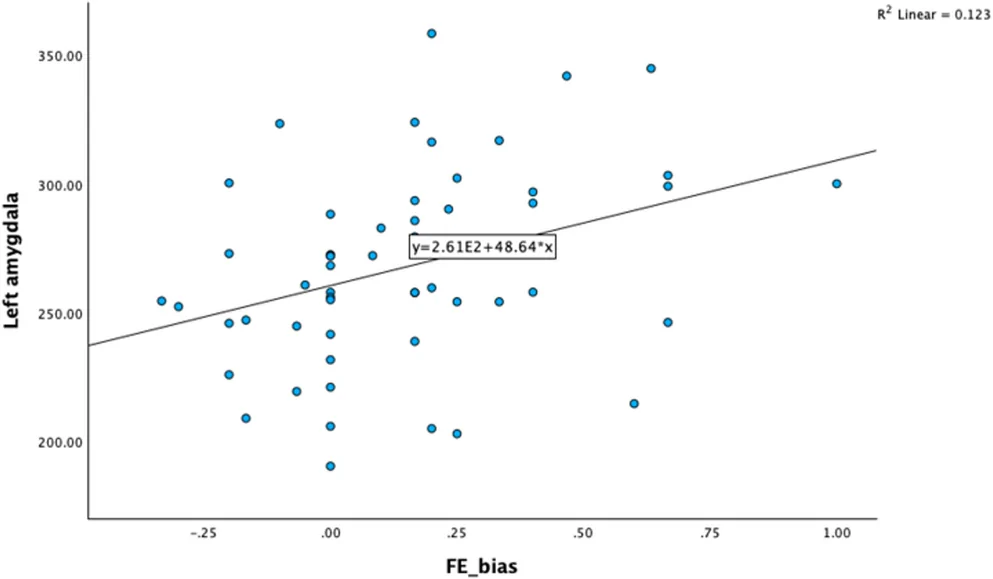
Left amygdala and fear bias scores at 30 months

At 30 months, the left amygdala volume adjusted for ICV correlated with FE_bias score after controlling for age at MRI scan (*β* = − 0.064, *p* =.025, *SE* = 0.027, 95% *CI* [–0.120, − 0.009]; model: *F*(2, 29) = 3.97, *p* =.030, *R²* = 0.215), indicating that FE_bias score and age at MRI scan explained approximately 21.5% of the variance in left amygdala volume. The regression equation was: Left amygdala = 0.458 − 0.064 (FE_bias) − 0.011(age at scan) (*M* = 0.13), but not with NE_bias (*β* = − 0.017, *p* =.667, *SE* = 0.038, 95% *CI* [–0.095, 0.062]) (*M* = 0.10) or HA_bias (*β* = − 0.037, *p* =.413, *SE* = 0.045, 95% *CI* [–0.129, 0.054]) (*M* = 0.01) (Fig. 4) (supplementary Table 5). No associations were observed with the right amygdala volume adjusted for ICV (supplementary Table 6). At 60 months, neither the left nor right amygdala volumes were associated with any of the emotion-specific bias scores. No significant sex interaction was observed for any emotion-specific bias scores at 30 or 60 months.

### The associations between the left and right amygdala MD and attention disengagement from faces and fear bias

**Fig. 5:**
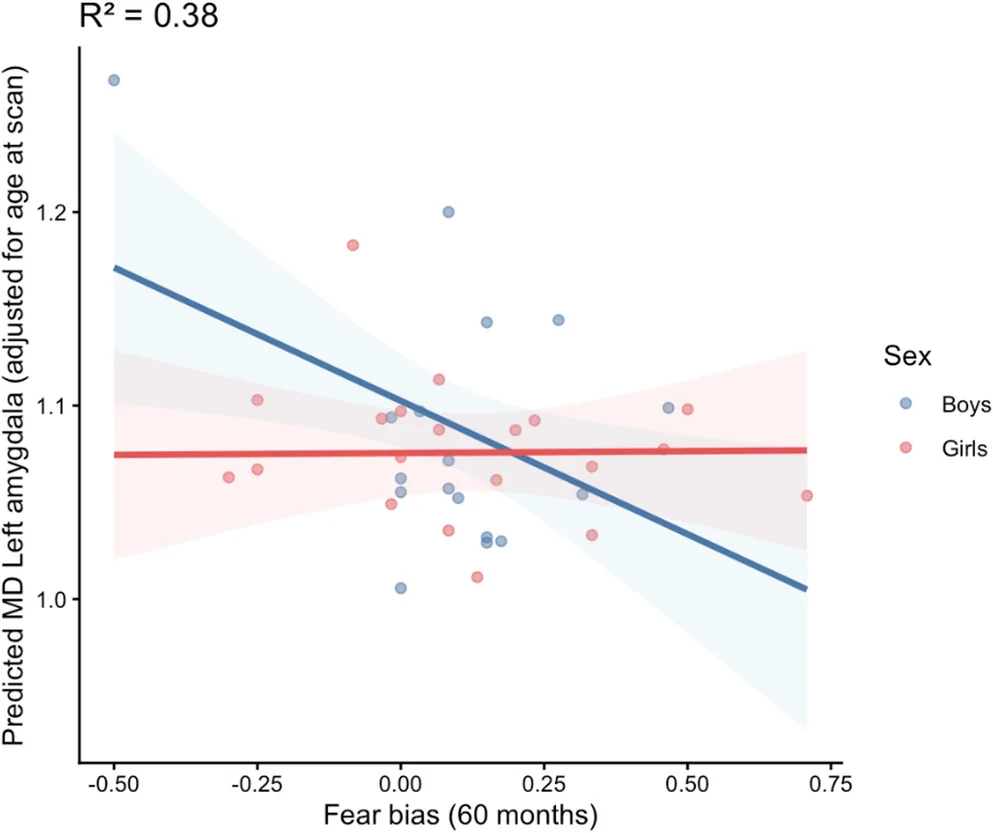
Interaction between fear bias and sex predicting left amygdala mean diffusivity (MD) at 60 months. Lines represent model-predicted MD values from the Fear bias × Sex model, adjusted for age at scan; shaded bands indicate 95% CIs

At 30 months, no significant overall or sex interaction effects were found between DPs in any condition as well as fear bias and MD in the bilateral amygdala. At 60 months, after controlling for age at scan time, there was a significant sex interaction between fear bias and MD in the left amygdala (*β* = −0.140, *p* =.046, *SE* = 0.067, 95% *CI* [− 0.277, − 0.003]; model: *F*(4, 32) = 4.98, *p* =.003, *R²* = 0.383), indicating that sex, fear bias, age at scan, and their interaction explained approximately 38.3% of the variance in left amygdala MD. The regression equation was:

MD left amygdala = 1.150 + 0.027 (sex) + 0.002 (Fear bias) − 0.020 (age ats can) − 0.140 (sex × Fear bias). Simple slopes (estimated marginal effects) derived from this interaction indicated a significant negative association between fear bias and MD in the left amygdala in boys (*β* = − 0.137, *p* =.018) but not in girls (*β* = 0.002 *p* =.961) (Fig. 5). All analyses, both significant and non-significant, are presented in the supplementary tables.

### The associations between the left and right amygdala MD and emotion-specific bias scores

No significant associations were observed between MD in the left or right amygdala and emotion-specific bias scores at either 30 or 60 month. Additionally, no sex-specific associations were identified, with all p-values exceeding the threshold for statistical significance. All analyses, both significant and non-significant, are presented in the supplementary tables.

## Discussion

The goal of this study was to investigate whether amygdala volume and diffusion characteristics measured at 2–8 weeks of age predict later fear processing, assessed through eye-tracking in children at 30 and 60 months. Building on our earlier work, we extended the analyses within a longitudinal birth cohort to examine how neonatal amygdala structure relates to fear processing beyond infancy.

Our findings suggest that associations between neonatal amygdala structure and fear-related attention may vary across early development. At 8 months, infants with larger amygdala volumes were more likely to disengage from fearful faces [26]. At 30 months, the pattern reversed, with larger left amygdala volumes linked to less disengagement from fearful faces. By 60 months, this relationship was no longer present. Fear processing patterns shift substantially during early development [58]. The tendency of infants to disengage less often from fearful vs. non-fearful faces when distracted by lateral salient stimuli peaks during the second half of the first year [59], and declines between 30 and 60 months [13, 60–62]. These age-dependent associations likely reflect developmental changes in the functional organization of emotion–attention systems rather than a static role of the amygdala.

This developmental shift likely reflects the amygdala’s evolving role within maturing emotion–attention networks [63, 64]. Longitudinal neuroimaging studies indicate that connectivity between the amygdala and the prefrontal cortex strengthens substantially from infancy through early childhood [65, 66]. Neurodevelopmental processes, including synaptic pruning and myelination, facilitate the refinement of long-range connectivity, between these regions over time [67]. As these regulatory networks mature, the behavioral relevance of neonatal amygdala structure may change, shifting from shaping early attentional reactivity to interacting with emerging top–down control processes [65, 66]. This interpretation aligns with evidence that in infancy the amygdala supports attentional flexibility within still-immature regulatory systems [68]. Consequently, the direction and strength of amygdala–fear bias associations may vary depending on whether a given fear bias profile is developmentally typical (as in infancy) or atypical (as in early childhood). These findings may reflect early-emerging individual differences in socioemotional processing rather than fixed behavioral tendencies [69, 70]. The shifting associations observed here suggest that early amygdala characteristics may index neurobiological predispositions whose behavioral expression evolves as emotion–attention systems mature. Consistent with this view, neonatal amygdala structure has been linked to later anxiety-related behaviors and self-regulation capacities [69–71]. Clinically, this implies that early neural variation may confer sensitivity to developmental context rather than stable risk.

In our DTI measures, our previous study at 8 months revealed that higher right amygdala MD was associated with lower DPs from fearful faces in the overall sample [27]. At 60 months, in boys, higher MD in the left amygdala was associated with a reduced fear bias. Sex differences in fear processing may help explain this male-specific pattern. Studies show that males exhibit reduced fear generalization compared to females, reflecting greater reliance on contextual processing mechanisms [72]. This suggests that boys may exhibit reduced fear bias not due to lower threat sensitivity, but because threat processing relies on partially distinct neural mechanisms during development. In this context, greater neonatal amygdala diffusivity was linked to higher fear bias in infancy but lower fear bias by early childhood. These results suggests that the behavioral significance of neonatal amygdala microstructure changes over time. In infancy, higher MD may reflect less mature microstructures associated with heightened emotional reactivity, resulting in greater attention to fearful stimuli. By early childhood, as regulatory systems mature, the same early microstructural characteristics may instead relate to more flexible or regulated threat processing. Thus, neonatal amygdala diffusivity may not confer a fixed risk or advantage but rather shape the trajectory of emotional attention across development. Together, the structural MRI and DTI findings suggest that both early amygdala macrostructure (volume) and microstructure (MD) may index neurodevelopmental variation in emotion–attention systems whose behavioral significance evolves across childhood. While volumetric differences may reflect early organizational properties related to attentional engagement with threat, microstructural characteristics may capture aspects of neural maturation that influence the flexibility of threat processing. The shifting associations observed across age therefore point to a common developmental mechanism in which early amygdala characteristics interact with emerging regulatory networks to shape socioemotional attention over time.

Importantly, these developmental processes unfold within environmental contexts that may further influence emerging emotional regulation [73, 74]. Factors such as prenatal stress exposure, postnatal caregiving quality, and maternal mental health have been linked to variation in amygdala development and may moderate how early neural characteristics relate to later socioemotional processing [69, 74–79]. Accordingly, neonatal amygdala structure may reflect early neurobiological predispositions whose behavioral expression evolves through ongoing interaction with both neural maturation and environmental experience. These early differences may therefore remain relevant for later socioemotional functioning even as their observable behavioral manifestations change over time.

This study has several strengths that enhance the robustness of our findings. First, the design allowed us to investigate whether neonatal amygdala characteristics predict fear processing at 30- and 60-months using eye-tracking, offering insights into the developmental trajectory of emotion–attention interactions. While the standard infant fear bias metric (fearful vs. neutral/happy faces) captures threat-biased attention in infancy, it may become less suitable in older children. Developmental studies demonstrate that affect-biased attention patterns change qualitatively between infancy and preschool; for example, fear bias declines nonlinearly between 8 and 60 months [13, 80]. Consequently, using emotion-specific bias scores at 30 and 60 months, instead of or together with a simple fearful versus neutral/happy contrast, may provide a more sensitive and age-appropriate measure of children’s attention to different emotional expressions. Second, combining structural and diffusion imaging offered complementary insight into the developmental and neurobiological foundations of later fear processing. Volumes reflect macrostructural features such as overall size, whereas MD is sensitive to microstructural properties including cell/neurite density, and extracellular water content [45, 81]. Higher MD in neonatal brain is often interpreted as reflecting less mature or less densely packed microstructure [81–83]. Considering both modalities together therefore provides complementary information: for example, larger structural volume paired with higher MD may suggest greater overall size but relatively immature microstructure, whereas larger volume with lower MD may indicate both growth and more advanced microstructural organization. Integrating these modalities provides a more comprehensive view of early amygdala organization [84].

Several limitations should be acknowledged. Although the sample size is substantial given the challenges of neonatal imaging and longitudinal follow-up, larger cohorts are needed to replicate these findings and explore potential moderating factors, such as genetic variability and environmental influences. Additionally, imaging data were only collected during infancy; future studies with multiple imaging time points across development would allow a more comprehensive understanding of how brain structure evolves in relation to emotional attention. Finally, as the analyses were exploratory, we did not apply correction for multiple comparisons. Accordingly, these findings should be interpreted with caution and considered hypothesis-generating rather than confirmatory. In conclusion, this study provides preliminary evidence that structural characteristics of the neonatal amygdala may be associated with later individual differences in attention to threat-related emotional cues. The variability in findings across developmental stages suggests that the relationship between early brain structure and socioemotional attention is dynamic and potentially influenced by postnatal experiences. These findings underscore the importance of early neural development in shaping emerging fear-related traits and highlight the need for longitudinal approaches to better understand their implications for later emotional and psychological functioning.

## Supplementary Information

Below is the link to the electronic supplementary material.

Supplementary File 1 (DOCX 145 KB)

## Data Availability

The data used in this study are available from the corresponding author upon request.
