# Supplementary material for: Neonatal amygdala and fear processing across early childhood
Source: Eur Child Adolesc Psychiatry. 2026 Apr 27;35(8):2607–20. doi: 10.1007/s00787-026-03041-3 (PMC13652908; doi:10.1007/s00787-026-03041-3)
Supplement: Supplementary file 1 [file 787_2026_3041_MOESM1_ESM.docx]

These are supplementary tables of the analyses. The notation "0a" indicates a parameter that has been set to zero due to redundancy in the model. For the sex variable, Sex 1 represents boys, and Sex 2 represents girls.

**Table 1.** Associations between newborn left amygdala volume (adjusted for ICV) and disengagement probaabilities (DPs) from fearful (FE), neutral (NE), happy (HA), and control (CS; non-face) pictures, as well as fear bias, at **30** months.

| Model | Parameter | B | Std. Error | t | p | 95% CI Lower | 95% CI Upper |
| --- | --- | --- | --- | --- | --- | --- | --- |
| DP_FE | Intercept | 0.502 | 0.031 | 16.371 | <.001 | 0.440 | 0.565 |
|  | **DP_FE30kk** | **-0.058** | 0.027 | -2.140 | **.041** | -0.114 | -0.003 |
|  | ageatscan | -0.009 | 0.006 | -1.449 | .158 | -0.022 | 0.004 |
| DP_HA | Intercept | 0.491 | 0.045 | 10.828 | <.001 | 0.398 | 0.584 |
|  | DP_HA30kk | -0.039 | 0.054 | -0.717 | .479 | -0.150 | 0.072 |
|  | ageatscan | -0.008 | 0.007 | -1.117 | .273 | -0.023 | 0.007 |
| DP_NE | Intercept | 0.473 | 0.044 | 10.755 | <.001 | 0.383 | 0.563 |
|  | DP_NE30kk | -0.010 | 0.040 | -0.243 | .810 | -0.091 | 0.071 |
|  | ageatscan | -0.010 | 0.007 | -1.432 | .163 | -0.024 | 0.004 |
| DP_CS | Intercept | 0.458 | 0.042 | 11.014 | <.001 | 0.373 | 0.543 |
|  | DP_CS30kk | 0.009 | 0.042 | 0.220 | .827 | -0.078 | 0.096 |
|  | ageatscan | -0.010 | 0.007 | -1.428 | .164 | -0.024 | 0.004 |
| Fearbias | Intercept | 0.455 | 0.025 | 17.883 | <.001 | 0.403 | 0.507 |
|  | **Fearbias30kk** | **0.073** | 0.033 | 2.238 | **.033** | 0.006 | 0.141 |
|  | ageatscan | -0.009 | 0.006 | -1.496 | .146 | -0.022 | 0.003 |

Table 2. Associations between newborn right amygdala volume (adjusted for ICV) and disengagement probaabilities (DPs) from fearful (FE), neutral (NE), happy (HA), and control (CS; non-face) pictures, as well as fear bias, at **30** months.

| Model | Parameter | B | Std. Error | t | p | 95% CI Lower | 95% CI Upper |
| --- | --- | --- | --- | --- | --- | --- | --- |
| DP_FE | Intercept | 0.489 | 0.033 | 14.789 | <.001 | 0.421 | 0.557 |
|  | DP_FE30kk | -0.044 | 0.029 | -1.482 | .149 | -0.104 | 0.017 |
|  | ageatscan | -0.010 | 0.007 | -1.460 | .155 | -0.024 | 0.004 |
| DP_HA | Intercept | 0.475 | 0.047 | 10.007 | <.001 | 0.378 | 0.572 |
|  | DP_HA30kk | -0.020 | 0.057 | -0.353 | .727 | -0.136 | 0.096 |
|  | ageatscan | -0.009 | 0.007 | -1.266 | .216 | -0.025 | 0.006 |
| DP_NE | Intercept | 0.466 | 0.046 | 10.179 | <.001 | 0.372 | 0.559 |
|  | DP_NE30kk | -0.005 | 0.041 | -0.127 | .900 | -0.089 | 0.079 |
|  | ageatscan | -0.010 | 0.007 | -1.451 | .157 | -0.025 | 0.004 |
| DP_CS | Intercept | 0.496 | 0.042 | 11.716 | <.001 | 0.410 | 0.583 |
|  | DP_CS30kk | -0.047 | 0.043 | -1.096 | .282 | -0.136 | 0.041 |
|  | ageatscan | -0.009 | 0.007 | -1.264 | .216 | -0.023 | 0.006 |
| Fearbias | Intercept | 0.453 | 0.027 | 16.546 | <.001 | 0.397 | 0.510 |
|  | Fearbias30kk | 0.058 | 0.035 | 1.627 | .114 | -0.015 | 0.130 |
|  | ageatscan | -0.010 | 0.007 | -1.495 | .146 | -0.024 | 0.004 |

Table 3. Associations between newborn left amygdala volume (adjusted for ICV), disengagement probaabilities (DPs) from fearful (FE), neutral (NE), happy (HA), and control (CS; non-face) pictures, as well as fear bias, at **30** months, with sex as an interaction term.

| Model | Parameter | B | Std. Error | t | p | 95% CI Lower | 95% CI Upper |
| --- | --- | --- | --- | --- | --- | --- | --- |
| DP_FE*Sex | Intercept | 2.560 | 0.330 | 7.693 | <.001 | 1.890 | 3.230 |
|  | DP_FE | -0.150 | 0.310 | -0.479 | .634 | -0.760 | 0.470 |
|  | [Sex=1] * DP_FE | -0.380 | 0.400 | -0.940 | .352 | -1.190 | 0.430 |
|  | [Sex=2] * DP_FE | 0a | . | . | . | . | . |
|  | [Sex=1] | 0.470 | 0.300 | 1.543 | .129 | -0.140 | 1.080 |
|  | [Sex=2] | 0a | . | . | . | . | . |
|  | ageatscan | 0.0036 | 0.0071 | 0.503 | .617 | -0.0107 | 0.0178 |
| DP_HA*Sex | Intercept | 2.340 | 0.410 | 5.717 | <.001 | 1.520 | 3.160 |
|  | DP_HA | 0.110 | 0.450 | 0.256 | .799 | -0.780 | 1.010 |
|  | [Sex=1] * DP_HA | -0.740 | 0.580 | -1.286 | .204 | -1.910 | 0.420 |
|  | [Sex=2] * DP_HA | 0a | . | . | . | . | . |
|  | [Sex=1] | 0.840 | 0.490 | 1.694 | .097 | -0.160 | 1.830 |
|  | [Sex=2] | 0a | . | . | . | . | . |
|  | ageatscan | 0.0043 | 0.0070 | 0.625 | .535 | -0.0096 | 0.0183 |
| DP_NE* Sex | Intercept | 2.460 | 0.300 | 8.250 | <.001 | 1.860 | 3.060 |
|  | DP_NE | 0.030 | 0.300 | 0.093 | .926 | -0.580 | 0.630 |
|  | [Sex=1] * DP_NE | 0.040 | 0.470 | 0.089 | .929 | -0.910 | 1.000 |
|  | [Sex=2] * DP_NE | 0a | . | . | . | . | . |
|  | [Sex=1] | 0.180 | 0.370 | 0.488 | .628 | -0.560 | 0.920 |
|  | [Sex=2] | 0a | . | . | . | . | . |
|  | ageatscan | 0.0024 | 0.0071 | 0.344 | .732 | -0.0118 | 0.0166 |
| DP_CS* Sex | Intercept | 2.380 | 0.350 | 6.854 | <.001 | 1.680 | 3.080 |
|  | DP_CS | 0.140 | 0.370 | 0.371 | .713 | -0.610 | 0.880 |
|  | [Sex=1] * DP_CS | -0.020 | 0.480 | -0.042 | .966 | -0.990 | 0.950 |
|  | [Sex=2] * DP_CS | 0a | . | . | . | . | . |
|  | [Sex=1] | 0.230 | 0.420 | 0.542 | .590 | -0.620 | 1.080 |
|  | [Sex=2] | 0a | . | . | . | . | . |
|  | ageatscan | 0.0018 | 0.0071 | 0.255 | .800 | -0.0124 | 0.0160 |
| Fearbias*Sex | Intercept | 2.470 | 0.190 | 12.837 | <.001 | 2.090 | 2.860 |
|  | Fearbias | 0.280 | 0.370 | 0.774 | .443 | -0.450 | 1.020 |
|  | [Sex=1] * Fearbias | 0.230 | 0.470 | 0.481 | .632 | -0.720 | 1.180 |
|  | [Sex=2] * Fearbias | 0a | . | . | . | . | . |
|  | [Sex=1] | 0.170 | 0.110 | 1.551 | .127 | -0.050 | 0.390 |
|  | [Sex=2] | 0a | . | . | . | . | . |
|  | ageatscan | 0.0022 | 0.0072 | 0.309 | .758 | -0.0122 | 0.0167 |

Table 4. Associations between newborn right amygdala volume (adjusted for ICV), disengagement probaabilities (DPs) from fearful (FE), neutral (NE), happy (HA), and control (CS; non-face) pictures, as well as fear bias, at **30** months, with sex as an interaction term.

| Model | Parameter | B | Std. Error | t | P | Lower Bound (95% CI) | Upper Bound (95% CI) |
| --- | --- | --- | --- | --- | --- | --- | --- |
| DP_FE*Sex | Intercept | 2.570 | 0.350 | 7.316 | <.001 | 1.860 | 3.280 |
|  | DP_FE | -0.120 | 0.320 | -0.381 | 0.704 | -0.770 | 0.530 |
|  | [Sex=1] * DP_FE | -0.110 | 0.430 | -0.254 | 0.801 | -0.960 | 0.750 |
|  | [Sex=2] * DP_FE | 0a | . | . | . | . | . |
|  | [Sex=1] | 0.360 | 0.320 | 1.115 | 0.270 | -0.290 | 1.000 |
|  | [Sex=2] | 0a | . | . | . | . | . |
|  | ageatscan | 0.0004 | 0.010 | 0.058 | 0.954 | -0.010 | 0.020 |
| DP_HA*Sex | Intercept | 2.370 | 0.430 | 5.583 | <.001 | 1.520 | 3.230 |
|  | DP_HA | 0.100 | 0.460 | 0.224 | 0.823 | -0.830 | 1.040 |
|  | [Sex=1] * DP_HA | -0.430 | 0.600 | -0.712 | 0.480 | -1.630 | 0.780 |
|  | [Sex=2] * DP_HA | 0a | . | . | . | . | . |
|  | [Sex=1] | 0.650 | 0.510 | 1.259 | 0.214 | -0.390 | 1.680 |
|  | [Sex=2] | 0a | . | . | . | . | . |
|  | ageatscan | 0.001 | 0.010 | 0.176 | 0.861 | -0.010 | 0.020 |
| DP_NE*Sex | Intercept | 2.270 | 0.300 | 7.544 | <.001 | 1.660 | 2.870 |
|  | DP_NE | 0.280 | 0.300 | 0.935 | 0.354 | -0.320 | 0.890 |
|  | [Sex=1] * DP_NE | -0.120 | 0.480 | -0.249 | 0.805 | -1.080 | 0.840 |
|  | [Sex=2] * DP_NE | 0a | . | . | . | . | . |
|  | [Sex=1] | 0.360 | 0.370 | 0.976 | 0.334 | -0.380 | 1.110 |
|  | [Sex=2] | 0a | . | . | . | . | . |
|  | ageatscan | 0.001 | 0.010 | 0.135 | 0.893 | -0.010 | 0.020 |
| DP_CS*Sex | Intercept | 2.430 | 0.350 | 6.870 | <.001 | 1.720 | 3.140 |
|  | DP_CS | 0.060 | 0.380 | 0.162 | 0.872 | -0.700 | 0.820 |
|  | [Sex=1] * DP_CS | -0.180 | 0.490 | -0.364 | 0.718 | -1.170 | 0.810 |
|  | [Sex=2] * DP_CS | 0a | . | . | . | . | . |
|  | [Sex=1] | 0.440 | 0.430 | 1.027 | 0.310 | -0.420 | 1.300 |
|  | [Sex=2] | 0a | . | . | . | . | . |
|  | ageatscan | 0.0003 | 0.010 | 0.046 | 0.964 | -0.010 | 0.010 |
| Fearbias*Sex | Intercept | 2.510 | 0.200 | 12.599 | <.001 | 2.110 | 2.920 |
|  | Fearbias | 0.440 | 0.380 | 1.165 | 0.250 | -0.320 | 1.200 |
|  | [Sex=1] * Fearbias | -0.200 | 0.490 | -0.402 | 0.689 | -1.180 | 0.790 |
|  | [Sex=2] * Fearbias | 0a | . | . | . | . | . |
|  | [Sex=1] | 0.280 | 0.110 | 2.491 | 0.016 | 0.050 | 0.510 |
|  | [Sex=2] | 0a | . | . | . | . | . |
|  | ageatscan | -0.002 | 0.010 | -0.222 | 0.826 | -0.020 | 0.010 |

Table 5. Associations between newborn left amygdala volume (adjusted for ICV) and emotion-specific bias scores (neutral bias, happy bias, and fearful bias), calculated by subtracting disengagement probabilities (DPs) in each condition from the scrambled-control picture, at **30** months.

| Model | Parameter | B | Std. Error | t | p | 95% CI Lower | 95% CI Upper |
| --- | --- | --- | --- | --- | --- | --- | --- |
| FE_bias | Intercept | 0.458 | 0.025 | 18.319 | <.001 | 0.407 | 0.510 |
|  | FE_bias | **–0.064** | 0.027 | –2.359 | **.025** | –0.120 | –0.009 |
|  | ageatscan | –0.011 | 0.006 | –1.755 | .090 | –0.024 | 0.002 |
| HA_bias | Intercept | 0.462 | 0.027 | 17.113 | <.001 | 0.407 | 0.518 |
|  | HA_bias | –0.037 | 0.045 | –0.830 | .413 | –0.129 | 0.054 |
|  | ageatscan | –0.009 | 0.007 | –1.350 | .187 | –0.023 | 0.005 |
| NE_bias | Intercept | 0.467 | 0.028 | 16.966 | <.001 | 0.411 | 0.523 |
|  | NE_bias | –0.017 | 0.038 | –0.435 | .667 | –0.095 | 0.062 |
|  | ageatscan | –0.011 | 0.007 | –1.479 | .150 | –0.025 | 0.004 |

Table 6. Associations between newborn right amygdala volume (adjusted for ICV) and emotion-specific bias scores (neutral bias, happy bias, and fearful bias), calculated by subtracting disengagement probabilities (DPs) in each condition from the scrambled-control picture, at **30** months.

| Model | Parameter | B | Std. Error | t | p | 95% CI Lower | 95% CI Upper |
| --- | --- | --- | --- | --- | --- | --- | --- |
| FE_bias30kk | Intercept | 0.459 | 0.028 | 16.310 | <.001 | 0.401 | 0.517 |
|  | FE_bias30kk | –0.021 | 0.031 | –0.699 | .490 | –0.084 | 0.041 |
|  | ageatscan | –0.011 | 0.007 | –1.524 | .138 | –0.025 | 0.004 |
| HA_bias30kk | Intercept | 0.464 | 0.028 | 16.550 | <.001 | 0.407 | 0.521 |
|  | HA_bias30kk | 0.040 | 0.046 | 0.863 | .395 | –0.055 | 0.135 |
|  | ageatscan | –0.011 | 0.007 | –1.540 | .134 | –0.025 | 0.004 |
| NE_bias30kk | Intercept | 0.457 | 0.028 | 16.114 | <.001 | 0.399 | 0.515 |
|  | NE_bias30kk | 0.034 | 0.039 | 0.857 | .398 | –0.047 | 0.114 |
|  | ageatscan | –0.008 | 0.007 | –1.107 | .277 | –0.023 | 0.007 |

Table 7. Associations between newborn left amygdala volume (adjusted for ICV) and emotion-specific bias scores (neutral bias, happy bias, and fearful bias), calculated by subtracting disengagement probabilities (DPs) in each condition from the scrambled-control picture, **30** months with sex as an interaction term.

| Model | Parameter | B | Std. Error | t | p | Lower Bound (95% CI) | Upper Bound (95% CI) |
| --- | --- | --- | --- | --- | --- | --- | --- |
| FE_bias*Sex | Intercept | 2.474 | 0.186 | 13.328 | <.001 | 2.101 | 2.848 |
|  | FE_bias | 0.236 | 0.291 | 0.811 | .421 | -0.349 | 0.821 |
|  | [Sex=1] * FE_bias | 0.353 | 0.384 | 0.920 | .362 | -0.419 | 1.125 |
|  | [Sex=2] * FE_bias | 0a | . | . | . | . | . |
|  | [Sex=1] | 0.138 | 0.112 | 1.223 | .227 | -0.088 | 0.364 |
|  | [Sex=2] | 0a | . | . | . | . | . |
|  | ageatscan | 0.002 | 0.007 | 0.251 | .803 | -0.012 | 0.016 |
| HA_bias*Sex | Intercept | 2.469 | 0.190 | 12.971 | <.001 | 2.087 | 2.852 |
|  | HA_bias | 0.053 | 0.360 | 0.147 | .884 | -0.671 | 0.776 |
|  | [Sex=1] * HA_bias | 0.338 | 0.446 | 0.757 | .452 | -0.559 | 1.234 |
|  | [Sex=2] * HA_bias | 0a | . | . | . | . | . |
|  | [Sex=1] | 0.205 | 0.104 | 1.970 | .054 | -0.004 | 0.414 |
|  | [Sex=2] | 0a | . | . | . | . | . |
|  | ageatscan | 0.003 | 0.007 | 0.420 | .676 | -0.011 | 0.017 |
| NE_bias*Sex | Intercept | 2.489 | 0.196 | 12.725 | <.001 | 2.096 | 2.882 |
|  | NE_bias | 0.044 | 0.252 | 0.176 | .861 | -0.462 | 0.550 |
|  | [Sex=1] * NE_bias | 0.022 | 0.399 | 0.054 | .957 | -0.781 | 0.824 |
|  | [Sex=2] * NE_bias | 0a | . | . | . | . | . |
|  | [Sex=1] | 0.214 | 0.114 | 1.878 | .066 | -0.015 | 0.443 |
|  | [Sex=2] | 0a | . | . | . | . | . |
|  | ageatscan | 0.002 | 0.007 | 0.266 | .791 | -0.012 | 0.016 |

Table 8. Associations between newborn right amygdala volume (adjusted for ICV) and emotion-specific bias scores (neutral bias, happy bias, and fearful bias), calculated by subtracting disengagement probabilities (DPs) in each condition from the scrambled-control picture, **30** months with sex as an interaction term.

| Model | Parameter | B | Std. Error | t | p | 95% CI Lower | 95% CI Upper |
| --- | --- | --- | --- | --- | --- | --- | --- |
| FE_bias*Sex | Intercept | 2.494 | 0.200 | 12.457 | <.001 | 2.092 | 2.897 |
|  | FE_bias | 0.165 | 0.314 | 0.527 | .601 | -0.465 | 0.796 |
|  | [Sex=1] * FE_bias | -0.018 | 0.414 | -0.044 | .965 | -0.851 | 0.815 |
|  | [Sex=2] * FE_bias | 0.000 | 0.000 | 0.000 | .000 | 0.000 | 0.000 |
|  | [Sex=1] | 0.280 | 0.121 | 2.312 | .025 | 0.037 | 0.524 |
|  | [Sex=2] | 0.000 | 0.000 | 0.000 | .000 | 0.000 | 0.000 |
|  | ageatscan | -0.001 | 0.008 | -0.103 | .919 | -0.016 | 0.015 |
| HA_bias*Sex | Intercept | 2.482 | 0.198 | 12.538 | <.001 | 2.084 | 2.880 |
|  | HA_bias | -0.009 | 0.374 | -0.024 | .981 | -0.761 | 0.734 |
|  | [Sex=1] * HA_bias | 0.081 | 0.464 | 0.175 | .861 | -0.851 | 1.013 |
|  | [Sex=2] * HA_bias | 0.000 | 0.000 | 0.000 | .000 | 0.000 | 0.000 |
|  | [Sex=1] | 0.287 | 0.108 | 2.652 | .011 | 0.069 | 0.504 |
|  | [Sex=2] | 0.000 | 0.000 | 0.000 | .000 | 0.000 | 0.000 |
|  | ageatscan | 0.000 | 0.007 | 0.054 | .957 | -0.014 | 0.015 |
| NE_bias*Sex | Intercept | 2.470 | 0.197 | 12.534 | <.001 | 2.074 | 2.867 |
|  | NE_bias | -0.173 | 0.254 | -0.684 | .497 | -0.683 | 0.337 |
|  | [Sex=1] * NE_bias | -0.067 | 0.403 | -0.166 | .869 | -0.876 | 0.742 |
|  | [Sex=2] * NE_bias | 0.000 | 0.000 | 0.000 | .000 | 0.000 | 0.000 |
|  | [Sex=1] | 0.290 | 0.115 | 2.522 | .015 | 0.059 | 0.520 |
|  | [Sex=2] | 0.000 | 0.000 | 0.000 | .000 | 0.000 | 0.000 |
|  | ageatscan | 0.002 | 0.007 | 0.230 | .819 | -0.013 | 0.016 |

Table 9. Associations between newborn left amygdala volume (adjusted for ICV) and disengagement probaabilities (DPs) from fearful (FE), neutral (NE), happy (HA), and control (CS; non-face) pictures, as well as fear bias, at **60** months.

| Model | Parameter | B | Std. Error | t | p | 95% CI Lower | 95% CI Upper |
| --- | --- | --- | --- | --- | --- | --- | --- |
| DP_FE | Intercept | 2.672 | 0.213 | 12.544 | <.001 | 2.244 | 3.099 |
|  | DP_FE | 0.103 | 0.205 | 0.503 | .617 | -0.309 | 0.515 |
|  | ageatscan | -0.024 | 0.465 | -0.522 | .604 | -0.118 | 0.069 |
| DP_HA | Intercept | 2.608 | 0.209 | 12.451 | <.001 | 2.187 | 3.029 |
|  | DP_HA | 0.257 | 0.242 | 1.066 | .292 | -0.228 | 0.743 |
|  | ageatscan | -0.040 | 0.484 | -0.826 | .413 | -0.137 | 0.057 |
| DP_NE | Intercept | 2.852 | 0.201 | 14.204 | <.001 | 2.449 | 3.255 |
|  | DP_NE | -0.261 | 0.222 | -1.176 | .245 | -0.707 | 0.185 |
|  | ageatscan | -0.007 | 0.483 | -0.138 | .890 | -0.104 | 0.090 |
| DP_CS | Intercept | 2.704 | 0.251 | 10.773 | <.001 | 2.200 | 3.208 |
|  | DP_CS | 0.041 | 0.254 | 0.160 | .873 | -0.469 | 0.551 |
|  | ageatscan | -0.025 | 0.472 | -0.536 | .595 | -0.120 | 0.069 |
| Fearbias | Intercept | 2.715 | 0.177 | 15.336 | <.001 | 2.360 | 3.071 |
|  | Fearbias | -0.146 | 0.224 | -0.649 | .519 | -0.597 | 0.305 |
|  | ageatscan | -0.015 | 0.485 | -0.309 | .759 | -0.113 | 0.083 |

**Table 10.** Associations between newborn right amygdala volume (adjusted for ICV) and disengagement probaabilities (DPs) from fearful (FE), neutral (NE), happy (HA), and control (CS; non-face) pictures, as well as fear bias, at **60** months.

| Model | Parameter | B | Std. Error | t | p | 95% CI Lower | 95% CI Upper |
| --- | --- | --- | --- | --- | --- | --- | --- |
| DP_FE | Intercept | 2.567 | 0.208 | 12.348 | <.001 | 2.150 | 2.985 |
|  | DP_FE | 0.134 | 0.200 | 0.669 | .507 | -0.268 | 0.536 |
|  | ageatscan | -0.008 | 0.454 | -0.187 | .852 | -0.099 | 0.083 |
| DP_HA | Intercept | 2.691 | 0.207 | 13.009 | <.001 | 2.276 | 3.106 |
|  | DP_HA | -0.092 | 0.239 | -0.385 | .702 | -0.571 | 0.387 |
|  | ageatscan | -0.003 | 0.478 | -0.054 | .957 | -0.099 | 0.093 |
| DP_NE | Intercept | 2.765 | 0.196 | 14.088 | <.001 | 2.371 | 3.159 |
|  | DP_NE | -0.260 | 0.217 | -1.198 | .237 | -0.695 | 0.176 |
|  | ageatscan | 0.009 | 0.472 | 0.192 | .849 | -0.086 | 0.104 |
| DP_CS | Intercept | 2.453 | 0.243 | 10.111 | <.001 | 1.966 | 2.940 |
|  | DP_CS | 0.274 | 0.246 | 1.116 | .270 | -0.219 | 0.767 |
|  | ageatscan | -0.016 | 0.456 | -0.356 | .723 | -0.108 | 0.075 |
| Fearbias | Intercept | 2.607 | 0.170 | 15.334 | <.001 | 2.266 | 2.948 |
|  | Fearbias | -0.331 | 0.216 | -1.534 | .131 | -0.764 | 0.102 |
|  | ageatscan | 0.012 | 0.465 | 0.267 | .791 | -0.081 | 0.106 |

Table 11. Associations between newborn left amygdala volume (adjusted for ICV) and disengagement probaabilities (DPs) from fearful (FE), neutral (NE), happy (HA), and control (CS; non-face) pictures, as well as fear bias at **60** months with sex as an interaction term.

| Model | Parameter | B | Std. Error | t | P | Lower Bound (95% CI) | Upper Bound (95% CI) |
| --- | --- | --- | --- | --- | --- | --- | --- |
| DP_FE* Sex | Intercept | 2.653 | 0.279 | 9.529 | <.001 | 2.094 | 3.213 |
|  | DP_FE | 0.066 | 0.325 | 0.204 | .840 | -0.587 | 0.719 |
|  | [Sex=1] * DP_FE | 0.020 | 0.432 | 0.046 | .964 | -0.849 | 0.889 |
|  | [Sex=2] * DP_FE | 0a | . | . | . | . | . |
|  | [Sex=1] | 0.091 | 0.274 | 0.332 | .741 | -0.459 | 0.641 |
|  | [Sex=2] | 0a | . | . | . | . | . |
|  | ageatscan | -0.028 | 0.481 | -0.590 | .558 | -0.125 | 0.068 |
| DP_HA* Sex | Intercept | 2.434 | 0.287 | 8.487 | <.001 | 1.857 | 3.010 |
|  | DP_HA | 0.426 | 0.341 | 1.249 | .218 | -0.259 | 1.111 |
|  | [Sex=1] * DP_HA | -0.300 | 0.465 | -0.644 | .522 | -1.235 | 0.635 |
|  | [Sex=2] * DP_HA | 0a | . | . | . | . | . |
|  | [Sex=1] | 0.325 | 0.344 | 0.945 | .350 | -0.367 | 1.016 |
|  | [Sex=2] | 0a | . | . | . | . | . |
|  | ageatscan | -0.042 | 0.491 | -0.847 | .401 | -0.140 | 0.057 |
| DP_NE* Sex | Intercept | 2.727 | 0.261 | 10.464 | <.001 | 2.203 | 3.251 |
|  | DP_NE | -0.196 | 0.287 | -0.681 | .499 | -0.773 | 0.382 |
|  | [Sex=1] * DP_NE | -0.245 | 0.445 | -0.550 | .585 | -1.140 | 0.650 |
|  | [Sex=2] * DP_NE | 0a | . | . | . | . | . |
|  | [Sex=1] | 0.295 | 0.327 | 0.902 | .371 | -0.362 | 0.951 |
|  | [Sex=2] | 0a | . | . | . | . | . |
|  | ageatscan | -0.002 | 0.501 | -0.041 | .968 | -0.103 | 0.099 |
| DP_CS* Sex | Intercept | 2.971 | 0.349 | 8.523 | <.001 | 2.271 | 3.672 |
|  | DP_CS | -0.301 | 0.363 | -0.828 | .412 | -1.031 | 0.429 |
|  | [Sex=1] * DP_CS | 0.599 | 0.536 | 1.117 | .269 | -0.478 | 1.676 |
|  | [Sex=2] * DP_CS | 0a | . | . | . | . | . |
|  | [Sex=1] | -0.373 | 0.444 | -0.840 | .405 | -1.266 | 0.520 |
|  | [Sex=2] | 0a | . | . | . | . | . |
|  | ageatscan | -0.043 | 0.491 | -0.881 | .383 | -0.142 | 0.055 |
| Fearbias*Sex | Intercept | 2.664 | 0.184 | 14.492 | <.001 | 2.295 | 2.934 |
|  | Fearbias | -0.009 | 0.299 | -0.030 | .976 | -0.610 | 0.593 |
|  | [Sex=1] * Fearbias | -0.257 | 0.437 | -0.588 | .559 | -1.136 | 0.622 |
|  | [Sex=2] * Fearbias | 0a | . | . | . | . | . |
|  | [Sex=1] | 0.129 | 0.114 | 1.125 | .266 | -0.101 | 0.358 |
|  | [Sex=2] | 0a | . | . | . | . | . |
|  | ageatscan | -0.021 | 0.491 | -0.420 | .676 | -0.119 | 0.078 |

Table 12. Associations between newborn left amygdala volume (adjusted for ICV) and disengagement probaabilities (DPs) from fearful (FE), neutral (NE), happy (HA), and control (CS; non-face) pictures, as well as fear bias a **60** months with sex as an interaction term.

| ****Model**** | ****Parameter**** | ****B**** | ****Std. Error**** | ****t**** | ****P**** | ****95% CI Lower**** | ****95% CI Upper**** |
| --- | --- | --- | --- | --- | --- | --- | --- |
| DP_FE*Sex | **Intercept** | 2.552 | 0.262 | 9.735 | <.001 | 2.025 | 3.079 |
|  | **DP_FE** | 0.236 | 0.306 | 0.077 | 0.939 | -0.591 | 0.639 |
|  | **[Sex=1] * DP_FE** | 0.098 | 0.407 | 0.242 | 0.810 | -0.719 | 0.916 |
|  | **[Sex=1]** | 0.157 | 0.258 | 0.607 | 0.546 | -0.362 | 0.675 |
|  | **ageatscan** | -0.018 | 0.453 | -0.408 | 0.685 | -0.109 | 0.073 |
| DP_HA*Sex | **Intercept** | 2.403 | 0.272 | 8.849 | <.001 | 1.857 | 2.949 |
|  | **DP_HA** | 0.171 | 0.323 | 0.531 | 0.598 | -0.477 | 0.820 |
|  | **[Sex=1] * DP_HA** | -0.461 | 0.441 | -1.044 | 0.302 | -1.346 | 0.425 |
|  | **[Sex=1]** | 0.542 | 0.326 | 1.664 | 0.102 | -0.113 | 1.197 |
|  | **ageatscan** | -0.007 | 0.465 | -0.150 | 0.882 | -0.100 | 0.087 |
| DP_NE*Sex | **Intercept** | 2.686 | 0.245 | 10.976 | <.001 | 2.194 | 3.178 |
|  | **DP_NE** | -0.311 | 0.270 | -1.152 | 0.255 | -0.853 | 0.231 |
|  | **[Sex=1] * DP_NE** | -0.032 | 0.418 | -0.076 | 0.940 | -0.873 | 0.809 |
|  | **[Sex=1]** | 0.260 | 0.307 | 0.847 | 0.401 | -0.357 | 0.876 |
|  | **ageatscan** | 0.005 | 0.470 | 0.117 | 0.908 | -0.089 | 0.100 |
| DP_CS*Sex | **Intercept** | 2.710 | 0.327 | 8.294 | <.001 | 2.053 | 3.367 |
|  | **DP_CS** | -0.110 | 0.340 | -0.322 | 0.749 | -0.794 | 0.574 |
|  | **[Sex=1] * DP_CS** | 0.582 | 0.503 | 1.158 | 0.252 | -0.428 | 1.591 |
|  | **[Sex=1]** | -0.264 | 0.416 | -0.633 | 0.529 | -1.100 | 0.573 |
|  | **ageatscan** | -0.036 | 0.461 | -0.779 | 0.440 | -0.128 | 0.057 |
| ****Fearbias*Sex**** | **Intercept** | 2.512 | 0.170 | 14.781 | <.001 | 2.171 | 2.854 |
|  | **Fearbias** | -0.133 | 0.277 | -0.480 | 0.634 | -0.689 | 0.423 |
|  | **[Sex=1] * Fearbias** | -0.343 | 0.404 | -0.850 | 0.399 | -1.156 | 0.469 |
|  | **[Sex=1]** | 0.242 | 0.106 | 2.289 | 0.026 | 0.030 | 0.454 |
|  | **ageatscan** | 0.001 | 0.454 | 0.030 | 0.977 | -0.090 | 0.093 |

Table 13. Associations between newborn left amygdala volume (adjusted for ICV) and emotion-specific bias scores (neutral bias, happy bias, and fearful bias), calculated by subtracting disengagement probabilities (DPs) in each condition from the scrambled-control picture, at **60** months.

| ****Model**** | ****Parameter**** | ****B**** | ****Std. Error**** | ****t**** | ****P**** | ****95% CI Lower**** | ****95% CI Upper**** |
| --- | --- | --- | --- | --- | --- | --- | --- |
| ****FE_bias**** | **Intercept** | 2.740 | 0.177 | 15.494 | <.001 | 2.385 | 3.095 |
|  | **FE_bias** | -0.065 | 0.189 | -0.342 | 0.734 | -0.443 | 0.314 |
|  | **ageatscan** | -0.023 | 0.468 | -0.476 | 0.636 | -0.116 | 0.072 |
| ****HA_bias**** | **Intercept** | 2.772 | 0.181 | 15.304 | <.001 | 2.408 | 3.135 |
|  | **HA_bias** | -0.176 | 0.217 | -0.810 | 0.422 | -0.612 | 0.260 |
|  | **ageatscan** | -0.030 | 0.468 | -0.637 | 0.527 | -0.124 | 0.064 |
| ****NE_bias**** | **Intercept** | 2.656 | 0.182 | 14.625 | <.001 | 2.291 | 3.021 |
|  | **NE_bias** | 0.307 | 0.227 | 1.358 | 0.180 | -0.147 | 0.762 |
|  | **ageatscan** | -0.013 | 0.465 | -0.270 | 0.789 | -0.106 | 0.081 |

Table 14. Associations between newborn right amygdala volume (adjusted for ICV) and emotion-specific bias scores (neutral bias, happy bias, and fearful bias), calculated by subtracting disengagement probabilities (DPs) in each condition from the scrambled-control picture, at **60** months.

| ****Model**** | ****Parameter**** | ****B**** | ****Std. Error**** | ****t**** | ****P**** | ****95% CI Lower**** | ****95% CI Upper**** |
| --- | --- | --- | --- | --- | --- | --- | --- |
| ****FE_bias**** | **Intercept** | 2.642 | 0.173 | 15.263 | <.001 | 2.294 | 2.989 |
|  | **FE_bias** | 0.038 | 0.185 | 0.208 | 0.836 | -0.332 | 0.409 |
|  | **ageatscan** | -0.009 | 0.458 | -0.203 | 0.840 | -0.101 | 0.083 |
| ****HA_bias**** | **Intercept** | 2.585 | 0.175 | 14.745 | <.001 | 2.233 | 2.937 |
|  | **HA_bias** | 0.276 | 0.211 | 1.314 | 0.195 | -0.146 | 0.699 |
|  | **ageatscan** | 0.001 | 0.453 | 0.018 | 0.986 | -0.090 | 0.092 |
| ****NE_bias**** | **Intercept** | 2.522 | 0.172 | 14.670 | <.001 | 2.177 | 2.867 |
|  | **NE_bias** | 0.498 | 0.214 | 2.324 | 0.126 | 0.068 | 0.929 |
|  | **ageatscan** | 0.010 | 0.441 | 0.236 | 0.814 | -0.078 | 0.099 |

Table 15. Associations between newborn left amygdala volume (adjusted for ICV) and emotion-specific bias scores (neutral bias, happy bias, and fearful bias), calculated by subtracting disengagement probabilities (DPs) in each condition from the scrambled-control picture, **60** months with sex as an interaction term.

| ****Model**** | ****Parameter**** | ****B**** | ****Std. Error**** | ****t**** | ****P**** | ****95% CI Lower**** | ****95% CI Upper**** |
| --- | --- | --- | --- | --- | --- | --- | --- |
| ****FE_bias*Sex**** | **Intercept** | 2.722 | 0.187 | 14.590 | <.001 | 2.347 | 3.097 |
|  | **FE_bias** | -0.184 | 0.255 | -0.721 | 0.474 | -0.695 | 0.328 |
|  | **[Sex=1] * FE_bias** | 0.246 | 0.379 | 0.650 | 0.519 | -0.516 | 1.009 |
|  | **[Sex=2] * FE_bias** | 0a | . | . | . | . | . |
|  | **[Sex=1]** | 0.056 | 0.132 | 0.427 | 0.671 | -0.209 | 0.322 |
|  | **[Sex=2]** | 0a | . | . | . | . | . |
|  | **ageatscan** | -0.027 | 0.472 | -0.574 | 0.569 | -0.122 | 0.068 |
| ****HA_bias*Sex**** | **Intercept** | 2.766 | 0.183 | 15.131 | <.001 | 2.398 | 3.133 |
|  | **HA_bias** | -0.493 | 0.287 | -1.722 | 0.091 | -1.069 | 0.082 |
|  | **[Sex=1] * HA_bias** | 0.609 | 0.444 | 1.373 | 0.176 | -0.282 | 1.500 |
|  | **[Sex=2] * HA_bias** | 0a | . | . | . | . | . |
|  | **[Sex=1]** | 0.069 | 0.115 | 0.600 | 0.551 | -0.162 | 0.300 |
|  | **[Sex=2]** | 0a | . | . | . | . | . |
|  | **ageatscan** | -0.043 | 0.467 | -0.922 | 0.361 | -0.137 | 0.051 |
| ****NE_bias*Sex**** | **Intercept** | 2.650 | 0.185 | 14.355 | <.001 | 2.279 | 3.021 |
|  | **NE_bias** | 0.011 | 0.295 | 0.038 | 0.970 | -0.582 | 0.604 |
|  | **[Sex=1] * NE_bias** | 0.654 | 0.445 | 1.468 | 0.148 | -0.241 | 1.549 |
|  | **[Sex=2] * NE_bias** | 0a | . | . | . | . | . |
|  | **[Sex=1]** | 0.023 | 0.113 | 0.205 | 0.839 | -0.207 | 0.251 |
|  | **[Sex=2]** | 0a | . | . | . | . | . |
|  | **ageatscan** | -0.017 | 0.462 | -0.370 | 0.713 | -0.110 | 0.076 |

Table 16. Associations between newborn right amygdala volume (adjusted for ICV) and emotion-specific bias scores (neutral bias, happy bias, and fearful bias), calculated by subtracting disengagement probabilities (DPs) in each condition from the scrambled-control picture, **60** months with sex as an interaction term.

| ****Model**** | ****Parameter**** | ****B**** | ****Std. Error**** | ****t**** | ****P**** | ****95% CI Lower**** | ****95% CI Upper**** |
| --- | --- | --- | --- | --- | --- | --- | --- |
| ****FE_bias*Sex**** | **Intercept** | 2.575 | 0.176 | 14.601 | <.001 | 2.220 | 2.929 |
|  | **FE_bias** | -0.062 | 0.241 | -0.259 | 0.796 | -0.546 | 0.421 |
|  | **[Sex=1] * FE_bias** | 0.186 | 0.358 | 0.519 | 0.606 | -0.534 | 0.906 |
|  | **[Sex=2] * FE_bias** | 0a | . | . | . | . | . |
|  | **[Sex=1]** | 0.179 | 0.125 | 1.433 | 0.158 | -0.072 | 0.430 |
|  | **[Sex=2]** | 0a | . | . | . | . | . |
|  | **ageatscan** | -0.018 | 0.446 | -0.395 | 0.695 | -0.107 | 0.072 |
| ****HA_bias*Sex**** | **Intercept** | 2.576 | 0.168 | 15.308 | <.001 | 2.237 | 2.913 |
|  | **HA_bias** | -0.192 | 0.264 | -0.726 | 0.471 | -0.722 | 0.338 |
|  | **[Sex=1] * HA_bias** | 0.895 | 0.408 | 2.193 | 0.068 | 0.075 | 1.715 |
|  | **[Sex=2] * HA_bias** | 0a | . | . | . | . | . |
|  | **[Sex=1]** | 0.104 | 0.106 | 0.981 | 0.331 | -0.109 | 0.316 |
|  | **[Sex=2]** | 0a | . | . | . | . | . |
|  | **ageatscan** | -0.019 | 0.430 | -0.436 | 0.664 | -0.105 | 0.068 |
| ****NE_bias*Sex**** | **Intercept** | 2.467 | 0.171 | 14.504 | <.001 | 2.125 | 2.809 |
|  | **NE_bias** | 0.277 | 0.272 | 1.017 | 0.314 | -0.270 | 0.823 |
|  | **[Sex=1] * NE_bias** | 0.443 | 0.411 | 1.078 | 0.286 | -0.383 | 1.268 |
|  | **[Sex=2] * NE_bias** | 0a | . | . | . | . | . |
|  | **[Sex=1]** | 0.154 | 0.105 | 1.472 | 0.147 | -0.056 | 0.364 |
|  | **[Sex=2]** | 0a | . | . | . | . | . |
|  | **ageatscan** | 0.015 | 0.426 | 0.035 | 0.972 | -0.084 | 0.087 |

**Table 17.** Associations between newborn left amygdala mean diffusivity (MD) and disengagement probaabilities (DPs) from fearful (FE), neutral (NE), happy (HA), and control (CS; non-face) pictures, as well as fear bias, at **30** months.

| Model | Parameter | B | Std. Error | t | P | 95% CI Lower | 95% CI Upper |
| --- | --- | --- | --- | --- | --- | --- | --- |
| FE_Dp | Intercept | 1.188 | 0.032 | 36.623 | <.001 | 1.122 | 1.253 |
|  | DP_FE | -0.032 | 0.027 | -1.168 | 0.250 | -0.086 | 0.023 |
|  | ageatscan | -0.022 | 0.006 | -3.478 | 0.001 | -0.035 | -0.009 |
| Dp_HA | Intercept | 1.159 | 0.039 | 29.462 | <.001 | 1.079 | 1.238 |
|  | DP_HA | 0.005 | 0.040 | 0.124 | 0.902 | -0.077 | 0.087 |
|  | ageatscan | -0.022 | 0.007 | -3.313 | 0.002 | -0.035 | -0.008 |
| Dp_NE | Intercept | 1.143 | 0.037 | 30.672 | <.001 | 1.067 | 1.218 |
|  | DP_NE | 0.022 | 0.031 | 0.720 | 0.476 | -0.041 | 0.085 |
|  | ageatscan | -0.021 | 0.007 | -3.164 | 0.003 | -0.034 | -0.007 |
| Dp_CS | Intercept | 1.174 | 0.036 | 32.217 | <.001 | 1.101 | 1.248 |
|  | DP_CS | -0.015 | 0.035 | -0.434 | 0.667 | -0.085 | 0.055 |
|  | ageatscan | -0.021 | 0.006 | -3.252 | 0.002 | -0.034 | -0.008 |
| Fearbias | Intercept | 1.161 | 0.024 | 48.794 | <.001 | 1.113 | 1.209 |
|  | Fearbias | 0.052 | 0.030 | 1.759 | 0.086 | -0.008 | 0.113 |
|  | ageatscan | -0.022 | 0.006 | -3.567 | <.001 | -0.035 | -0.010 |

**Table 18.** Associations between newborn right amygdala mean diffusivity (MD) and disengagement probaabilities (DPs) from fearful (FE), neutral (NE), happy (HA), and control (CS; non-face) pictures, as well as fear bias, at **30** months.

| Model | Parameter | B | Std. Error | t | P | 95% CI Lower | 95% CI Upper |
| --- | --- | --- | --- | --- | --- | --- | --- |
| DP_FE | Intercept | 1.132 | 0.035 | 32.495 | <.001 | 1.061 | 1.202 |
|  | DP_FE | -0.008 | 0.029 | -0.269 | 0.789 | -0.066 | 0.051 |
|  | ageatscan | -0.007 | 0.007 | -0.983 | 0.332 | -0.021 | 0.007 |
| DP_HA | Intercept | 1.123 | 0.042 | 27.024 | <.001 | 1.039 | 1.207 |
|  | DP_HA | 0.003 | 0.043 | 0.071 | 0.944 | -0.083 | 0.089 |
|  | ageatscan | -0.007 | 0.007 | -0.962 | 0.342 | -0.021 | 0.007 |
| DP_NE | Intercept | 1.110 | 0.039 | 28.114 | <.001 | 1.030 | 1.189 |
|  | DP_NE | 0.018 | 0.033 | 0.533 | 0.597 | -0.049 | 0.084 |
|  | ageatscan | -0.006 | 0.007 | -0.852 | 0.399 | -0.020 | 0.008 |
| DP_CS | Intercept | 1.101 | 0.038 | 28.798 | <.001 | 1.024 | 1.178 |
|  | DP_CS | 0.031 | 0.036 | 0.861 | 0.394 | -0.042 | 0.105 |
|  | ageatscan | -0.007 | 0.007 | -1.071 | 0.291 | -0.021 | 0.006 |
| Fearbias | Intercept | 1.125 | 0.026 | 43.269 | <.001 | 1.072 | 1.177 |
|  | Fearbias | 0.019 | 0.033 | 0.595 | 0.556 | -0.046 | 0.085 |
|  | ageatscan | -0.007 | 0.007 | -1.003 | 0.322 | -0.021 | 0.007 |

Table 19. Associations between newborn left amygdala mean diffusivity (MD) and disengagement probaabilities (DPs) from fearful (FE), neutral (NE), happy (HA), and control (CS; non-face) pictures, as well as fear bias, at **30** months, with sex as an interaction term.

| Model | Parameter | B | Std. Error | t | P | 95% CI Lower | 95% CI Upper |
| --- | --- | --- | --- | --- | --- | --- | --- |
| DP_FE*Sex | Intercept | 1.173 | 0.052 | 22.734 | <.001 | 1.069 | 1.278 |
|  | DP_FE | -0.022 | 0.046 | -0.481 | 0.633 | -0.115 | 0.071 |
|  | [sex=1] * DP_FE | -0.012 | 0.061 | -0.194 | 0.847 | -0.136 | 0.112 |
|  | [sex=2] * DP_FE | 0a | . | . | . | . | . |
|  | [sex=1] | 0.018 | 0.047 | 0.391 | 0.698 | -0.077 | 0.113 |
|  | [sex=2] | 0a | . | . | . | . | . |
|  | ageatscan | -0.022 | 0.007 | -3.043 | 0.004 | -0.036 | -0.007 |
| DP_HA*Sex | Intercept | 1.230 | 0.058 | 21.245 | <.001 | 1.113 | 1.347 |
|  | DP_HA | -0.083 | 0.062 | -1.329 | 0.192 | -0.209 | 0.043 |
|  | [sex=1] * DP_HA | 0.148 | 0.080 | 1.854 | 0.072 | -0.014 | 0.310 |
|  | [sex=2] * DP_HA | 0a | . | . | . | . | . |
|  | [sex=1] | -0.113 | 0.069 | -1.642 | 0.109 | -0.252 | 0.026 |
|  | [sex=2] | 0a | . | . | . | . | . |
|  | ageatscan | -0.022 | 0.006 | -3.529 | 0.001 | -0.035 | -0.010 |
| DP_NE*Sex | Intercept | 1.139 | 0.043 | 26.255 | <.001 | 1.051 | 1.227 |
|  | DP_NE | 0.020 | 0.042 | 0.472 | 0.640 | -0.065 | 0.104 |
|  | [sex=1] * DP_NE | 0.003 | 0.063 | 0.054 | 0.957 | -0.125 | 0.131 |
|  | [sex=2] * DP_NE | 0a | . | . | . | . | . |
|  | [sex=1] | 0.009 | 0.050 | 0.170 | 0.866 | -0.093 | 0.111 |
|  | [sex=2] | 0a | . | . | . | . | . |
|  | ageatscan | -0.021 | 0.007 | -3.122 | 0.003 | -0.034 | -0.007 |
| DP_CS*Sex | Intercept | 1.170 | 0.051 | 22.821 | <.001 | 1.066 | 1.273 |
|  | DP_CS | -0.017 | 0.054 | -0.312 | 0.757 | -0.126 | 0.093 |
|  | [sex=1] * DP_CS | 0.3004 | 0.071 | 0.050 | 0.961 | -0.140 | 0.147 |
|  | [sex=2] * DP_CS | 0a | . | . | . | . | . |
|  | [sex=1] | 0.009 | 0.063 | 0.136 | 0.892 | -0.119 | 0.136 |
|  | [sex=2] | 0a | . | . | . | . | . |
|  | ageatscan | -0.021 | 0.007 | -3.204 | 0.003 | -0.035 | -0.008 |
| Fearbias*Sex | Intercept | 1.148 | 0.026 | 43.343 | <.001 | 1.094 | 1.201 |
|  | Fearbias | 0.011 | 0.047 | 0.224 | 0.824 | -0.085 | 0.106 |
|  | [sex=1] * Fearbias | 0.072 | 0.066 | 1.093 | 0.281 | -0.062 | 0.206 |
|  | [sex=2] * Fearbias | 0a | . | . | . | . | . |
|  | [sex=1] | 0.003 | 0.015 | 0.167 | 0.868 | -0.028 | 0.033 |
|  | [sex=2] | 0a | . | . | . | . | . |
|  | ageatscan | -0.019 | 0.007 | -2.822 | 0.008 | -0.033 | -0.005 |

Table 20. Associations between newborn right amygdala mean diffusivity (MD) and disengagement probaabilities (DPs) from fearful (FE), neutral (NE), happy (HA), and control (CS; non-face) pictures, as well as fear bias, at **30** months, with sex as an interaction term.

| Model | Parameter | B | Std. Error | t | P | 95% CI Lower | 95% CI Upper |
| --- | --- | --- | --- | --- | --- | --- | --- |
| DP_FE*Sex | Intercept | 1.149 | 0.055 | 20.780 | <.001 | 1.037 | 1.261 |
|  | DP_FE | -0.028 | 0.049 | -0.578 | 0.567 | -0.128 | 0.071 |
|  | [sex=1] * DP_FE | 0.038 | 0.066 | 0.579 | 0.566 | -0.095 | 0.171 |
|  | [sex=2] * DP_FE | 0a | . | . | . | . | . |
|  | [sex=1] | -0.019 | 0.050 | -0.376 | 0.709 | -0.121 | 0.083 |
|  | [sex=2] | 0a | . | . | . | . | . |
|  | ageatscan | -0.009 | 0.008 | -1.119 | 0.270 | -0.024 | 0.007 |
| DP_HA*Sex | Intercept | 1.187 | 0.062 | 19.066 | <.001 | 1.061 | 1.313 |
|  | DP_HA | -0.075 | 0.067 | -1.120 | 0.270 | -0.210 | 0.061 |
|  | [sex=1] * DP_HA | 0.131 | 0.086 | 1.529 | 0.135 | -0.043 | 0.305 |
|  | [sex=2] * DP_HA | 0a | . | . | . | . | . |
|  | [sex=1] | -0.101 | 0.074 | -1.372 | 0.178 | -0.251 | 0.048 |
|  | [sex=2] | 0a | . | . | . | . | . |
|  | ageatscan | -0.007 | 0.007 | -1.077 | 0.288 | -0.021 | 0.006 |
| DP_NE*Sex | Intercept | 1.120 | 0.046 | 24.427 | <.001 | 1.027 | 1.213 |
|  | DP_NE | -0.002 | 0.044 | -0.054 | 0.957 | -0.092 | 0.087 |
|  | [sex=1] * DP_NE | 0.044 | 0.067 | 0.665 | 0.510 | -0.091 | 0.180 |
|  | [sex=2] * DP_NE | 0a | . | . | . | . | . |
|  | [sex=1] | -0.025 | 0.053 | -0.472 | 0.639 | -0.133 | 0.083 |
|  | [sex=2] | 0a | . | . | . | . | . |
|  | ageatscan | -0.006 | 0.007 | -0.844 | 0.404 | -0.020 | 0.008 |
| DP_CS*Sex | Intercept | 1.095 | 0.054 | 20.300 | <.001 | 0.986 | 1.204 |
|  | DP_CS | 0.033 | 0.057 | 0.572 | 0.571 | -0.083 | 0.148 |
|  | [sex=1] * DP_CS | -0.002 | 0.075 | -0.022 | 0.982 | -0.153 | 0.150 |
|  | [sex=2] * DP_CS | 0a | . | . | . | . | . |
|  | [sex=1] | 0.011 | 0.066 | 0.161 | 0.873 | -0.123 | 0.145 |
|  | [sex=2] | 0a | . | . | . | . | . |
|  | ageatscan | -0.007 | 0.007 | -1.058 | 0.297 | -0.021 | 0.007 |
| Fearbias*Sex | Intercept | 1.118 | 0.029 | 38.046 | <.001 | 1.058 | 1.177 |
|  | Fearbias | 0.005 | 0.052 | 0.093 | 0.927 | -0.101 | 0.110 |
|  | [sex=1] * Fearbias | 0.022 | 0.073 | 0.301 | 0.765 | -0.126 | 0.170 |
|  | [sex=2] * Fearbias | 0a | . | . | . | . | . |
|  | [sex=1] | 0.006 | 0.017 | 0.365 | 0.718 | -0.028 | 0.040 |
|  | [sex=2] | 0a | . | . | . | . | . |
|  | ageatscan | -0.006 | 0.008 | -0.784 | 0.438 | -0.021 | 0.009 |

Table 21. Associations between newborn left amygdala mean diffusivity (MD) and emotion-specific bias scores (neutral bias, happy bias, and fearful bias), calculated by subtracting disengagement probabilities (DPs) in each condition from the scrambled-control picture at **30** months.

| Model | Parameter | B | Std. Error | t | P | 95% CI Lower | 95% CI Upper |
| --- | --- | --- | --- | --- | --- | --- | --- |
| ****FE_bias**** | Intercept | 1.163 | 0.025 | 47.447 | <.001 | 1.114 | 1.213 |
|  | FE_bias | -0.020 | 0.026 | -0.781 | .440 | -0.073 | 0.032 |
|  | ageatscan | -0.022 | 0.007 | -3.439 | .001 | -0.036 | -0.009 |
| ****HA_bias**** | Intercept | 1.163 | 0.025 | 47.218 | <.001 | 1.113 | 1.213 |
|  | HA_bias | 0.015 | 0.031 | 0.483 | .632 | -0.048 | 0.077 |
|  | ageatscan | -0.021 | 0.006 | -3.332 | .002 | -0.034 | -0.008 |
| ****NE_bias**** | Intercept | 1.160 | 0.025 | 47.052 | <.001 | 1.110 | 1.209 |
|  | NE_bias | 0.025 | 0.026 | 0.953 | .346 | -0.028 | 0.079 |
|  | ageatscan | -0.020 | 0.007 | -3.026 | .004 | -0.033 | -0.007 |

Table 22. Associations between newborn right amygdala mean diffusivity (MD) and emotion- specific bias scores (neutral bias, happy bias, and fearful bias), calculated by subtracting disengagement probabilities (DPs) in each condition from the scrambled-control picture at **30** months.

| Model | Parameter | B | Std. Error | t | P | 95% CI Lower | 95% CI Upper |
| --- | --- | --- | --- | --- | --- | --- | --- |
| **FE_bias** | Intercept | 1.126 | 0.026 | 43.602 | <.001 | 1.074 | 1.178 |
|  | FE_bias | -0.025 | 0.028 | -0.914 | 0.367 | -0.081 | 0.031 |
|  | ageatscan | -0.008 | 0.007 | -1.122 | 0.269 | -0.022 | 0.006 |
| **HA_bias** | Intercept | 1.125 | 0.026 | 43.394 | <.001 | 1.073 | 1.178 |
|  | HA_bias | -0.023 | 0.033 | -0.709 | 0.482 | -0.089 | 0.043 |
|  | ageatscan | -0.007 | 0.007 | -0.973 | 0.337 | -0.020 | 0.007 |
| **NE_bias** | Intercept | 1.126 | 0.026 | 42.785 | <.001 | 1.073 | 1.179 |
|  | NE_bias | -0.006 | 0.028 | -0.202 | 0.841 | -0.063 | 0.051 |
|  | ageatscan | -0.007 | 0.007 | -0.981 | 0.332 | -0.021 | 0.007 |

Table 23. Associations between newborn left amygdala mean diffusivity (MD) and emotion- specific bias scores (neutral bias, happy bias, and fearful bias), calculated by subtracting disengagement probabilities (DPs) in each condition from the scrambled-control picture at **30** months with sex as an interaction term.

| Model | Parameter | B | Std. Error | t | P | 95% CI Lower | 95% CI Upper |
| --- | --- | --- | --- | --- | --- | --- | --- |
| ****FE_bias*****Sex | Intercept | 1.156 | 0.027 | 42.507 | <.001 | 1.101 | 1.211 |
|  | FE_bias | -0.009 | 0.043 | -0.218 | 0.829 | -0.096 | 0.077 |
|  | [sex=1] * FE_bias | -0.016 | 0.058 | -0.270 | 0.789 | -0.132 | 0.101 |
|  | [sex=2] * FE_bias | 0a | . | . | . | . | . |
|  | [sex=1] | 0.008 | 0.017 | 0.498 | 0.622 | -0.026 | 0.043 |
|  | [sex=2] | 0a | . | . | . | . | . |
|  | ageatscan | -0.022 | 0.007 | -2.989 | 0.005 | -0.036 | -0.007 |
| ****HA_bias*****Sex | Intercept | 1.159 | 0.026 | 44.951 | <.001 | 1.106 | 1.211 |
|  | HA_bias | -0.035 | 0.049 | -0.725 | 0.473 | -0.135 | 0.064 |
|  | [sex=1] * HA_bias | 0.085 | 0.063 | 1.346 | 0.187 | -0.043 | 0.213 |
|  | [sex=2] * HA_bias | 0a | . | . | . | . | . |
|  | [sex=1] | 0.013 | 0.015 | 0.919 | 0.364 | -0.016 | 0.043 |
|  | [sex=2] | 0a | . | . | . | . | . |
|  | ageatscan | -0.022 | 0.006 | -3.470 | 0.001 | -0.035 | -0.009 |
| ****NE_bias*****Sex | Intercept | 1.153 | 0.027 | 43.501 | <.001 | 1.099 | 1.207 |
|  | NE_bias | 0.020 | 0.034 | 0.598 | 0.553 | -0.048 | 0.088 |
|  | [sex=1] * NE_bias | 0.011 | 0.054 | 0.197 | 0.845 | -0.099 | 0.120 |
|  | [sex=2] * NE_bias | 0a | . | . | . | . | . |
|  | [sex=1] | 0.012 | 0.016 | 0.766 | 0.449 | -0.020 | 0.044 |
|  | [sex=2] | 0a | . | . | . | . | . |
|  | ageatscan | -0.020 | 0.007 | -2.981 | 0.005 | -0.034 | -0.006 |

Table 24. Associations between newborn right amygdala mean diffusivity (MD) and emotion- specific bias scores (neutral bias, happy bias, and fearful bias), calculated by subtracting disengagement probabilities (DPs) in each condition from the scrambled-control picture at **30** months with sex as an interaction term.

| Model | Parameter | B | Std. Error | t | P | 95% CI Lower | 95% CI Upper |
| --- | --- | --- | --- | --- | --- | --- | --- |
| ****FE_bias*****Sex | Intercept | 1.126 | 0.029 | 39.301 | <.001 | 1.068 | 1.184 |
|  | FE_bias | -0.043 | 0.045 | -0.956 | 0.345 | -0.134 | 0.048 |
|  | [sex=1] * FE_bias | 0.034 | 0.061 | 0.554 | 0.583 | -0.089 | 0.157 |
|  | [sex=2] * FE_bias | 0a | . | . | . | . | . |
|  | [sex=1] | 0.012 | 0.018 | 0.683 | 0.499 | -0.024 | 0.048 |
|  | [sex=2] | 0a | . | . | . | . | . |
|  | ageatscan | -0.009 | 0.008 | -1.228 | 0.227 | -0.025 | 0.006 |
| ****HA_bias*****Sex | Intercept | 1.122 | 0.027 | 41.047 | <.001 | 1.067 | 1.178 |
|  | HA_bias | -0.073 | 0.052 | -1.402 | 0.169 | -0.178 | 0.032 |
|  | [sex=1] * HA_bias | 0.083 | 0.067 | 1.246 | 0.221 | -0.052 | 0.219 |
|  | [sex=2] * HA_bias | 0a | . | . | . | . | . |
|  | [sex=1] | 0.010 | 0.015 | 0.659 | 0.514 | -0.021 | 0.042 |
|  | [sex=2] | 0a | . | . | . | . | . |
|  | ageatscan | -0.007 | 0.007 | -1.085 | 0.285 | -0.021 | 0.006 |
| ****NE_bias*****Sex | Intercept | 1.120 | 0.028 | 39.501 | <.001 | 1.062 | 1.177 |
|  | NE_bias | -0.015 | 0.036 | -0.404 | 0.689 | -0.088 | 0.059 |
|  | [sex=1] * NE_bias | 0.021 | 0.058 | 0.369 | 0.714 | -0.095 | 0.138 |
|  | [sex=2] * NE_bias | 0a | . | . | . | . | . |
|  | [sex=1] | 0.011 | 0.017 | 0.675 | 0.504 | -0.023 | 0.045 |
|  | [sex=2] | 0a | . | . | . | . | . |
|  | ageatscan | -0.007 | 0.007 | -0.964 | 0.341 | -0.021 | 0.008 |

Table 25. Associations between newborn left amygdala mean diffusivity (MD) and disengagement probaabilities (DPs) from fearful (FE), neutral (NE), happy (HA), and control (CS; non-face) pictures, as well as fear bias, at **60** months.

| Model | Parameter | B | Std. Error | t | P | 95% CI Lower | 95% CI Upper |
| --- | --- | --- | --- | --- | --- | --- | --- |
| ****DP_FE**** | Intercept | 0.001 | 2.886E-5 | 39.732 | <.001 | 0.001 | 0.001 |
|  | DP_FE | 2.345E-5 | 3.002E-5 | 0.781 | 0.440 | -3.756E-5 | 8.447E-5 |
|  | ageatscan | -2.158E-5 | 6.369E-6 | -3.388 | 0.002 | -3.452E-5 | -8.632E-6 |
| ****DP_HA**** | Intercept | 0.001 | 2.840E-5 | 41.169 | <.001 | 0.001 | 0.001 |
|  | DP_HA | -2.307E-5 | 3.298E-5 | -0.699 | 0.489 | -9.009E-5 | 4.396E-5 |
|  | ageatscan | -1.977E-5 | 6.704E-6 | -2.949 | 0.006 | -3.340E-5 | -6.148E-6 |
| ****DP_NE**** | Intercept | 0.001 | 2.859E-5 | 40.634 | <.001 | 0.001 | 0.001 |
|  | DP_NE | -6.021E-6 | 3.131E-5 | -0.192 | 0.849 | -6.965E-5 | 5.761E-5 |
|  | ageatscan | -2.090E-5 | 6.652E-6 | -3.142 | 0.003 | -3.442E-5 | -7.381E-6 |
| ****DP_CS**** | Intercept | 0.001 | 3.584E-5 | 32.625 | <.001 | 0.001 | 0.001 |
|  | DP_CS | -1.460E-5 | 3.696E-5 | -0.395 | 0.695 | -8.971E-5 | 6.051E-5 |
|  | ageatscan | -2.099E-5 | 6.429E-6 | -3.264 | 0.003 | -3.405E-5 | -7.921E-6 |
| ****Fearbias**** | Intercept | 0.001 | 2.386E-5 | 48.455 | <.001 | 0.001 | 0.001 |
|  | Fearbias | -4.759E-5 | 3.393E-5 | -1.403 | 0.170 | 0.000 | 2.136E-5 |
|  | ageatscan | -1.905E-5 | 6.427E-6 | -2.963 | 0.006 | -3.211E-5 | -5.985E-6 |

Table 26. Associations between newborn right amygdala mean diffusivity (MD) and disengagement probaabilities (DPs) from fearful (FE), neutral (NE), happy (HA), and control (CS; non-face) pictures, as well as fear bias, at **60** months.

| Model | Parameter | B | Std. Error | t | P | 95% CI Lower | 95% CI Upper |
| --- | --- | --- | --- | --- | --- | --- | --- |
| ****DP_FE**** | Intercept | 0.001 | 3.228E-5 | 34.529 | <.001 | 0.001 | 0.001 |
|  | DP_FE | 1.455E-5 | 3.357E-5 | 0.433 | 0.667 | -5.368E-5 | 8.278E-5 |
|  | ageatscan | -6.919E-6 | 7.122E-6 | -0.971 | 0.338 | -2.139E-5 | 7.555E-6 |
| ****DP_HA**** | Intercept | 0.001 | 3.179E-5 | 35.250 | <.001 | 0.001 | 0.001 |
|  | DP_HA | 3.109E-6 | 3.691E-5 | 0.084 | 0.933 | -7.190E-5 | 7.812E-5 |
|  | ageatscan | -6.910E-6 | 7.503E-6 | -0.921 | 0.364 | -2.216E-5 | 8.338E-6 |
| ****DP_NE**** | Intercept | 0.001 | 3.173E-5 | 35.545 | <.001 | 0.001 | 0.001 |
|  | DP_NE | -1.263E-5 | 3.475E-5 | -0.363 | 0.718 | -8.325E-5 | 5.799E-5 |
|  | ageatscan | -5.992E-6 | 7.382E-6 | -0.812 | 0.423 | -2.099E-5 | 9.011E-6 |
| ****DP_CS**** | Intercept | 0.001 | 3.992E-5 | 28.039 | <.001 | 0.001 | 0.001 |
|  | DP_CS | 3.696E-6 | 4.117E-5 | 0.090 | 0.929 | -7.996E-5 | 8.735E-5 |
|  | ageatscan | -6.776E-6 | 7.161E-6 | -0.946 | 0.351 | -2.133E-5 | 7.776E-6 |
| ****Fearbias**** | Intercept | 0.001 | 2.710E-5 | 41.345 | <.001 | 0.001 | 0.001 |
|  | Fearbias | -2.543E-5 | 3.854E-5 | -0.660 | 0.514 | 0.000 | 5.288E-5 |
|  | ageatscan | -5.538E-6 | 7.300E-6 | -0.759 | 0.453 |  |  |

Table 27. Associations between newborn left amygdala mean diffusivity (MD) and disengagement probaabilities (DPs) from fearful (FE), neutral (NE), happy (HA), and control (CS; non-face) pictures, as well as fear bias, at **60** months, with sex as an interaction term.

| Model | Parameter | B (×1000) | Std. Error | t | P | 95% CI Lower | 95% CI Upper |
| --- | --- | --- | --- | --- | --- | --- | --- |
| ****DP_FE*Sex**** | Intercept | 1.000 | 0.038 | 30.099 | <.001 | 1.000 | 1.000 |
|  | DP_FE | 0.0215 | 0.043 | 0.503 | 0.618 | -0.0669 | 0.000 |
|  | [Sex=1] * DP_FE | 0.00236 | 0.064 | 0.037 | 0.971 | 0.000 | 0.000 |
|  | [Sex=1] | 0.0127 | 0.039 | 0.322 | 0.750 | -0.0677 | 0.0931 |
|  | [Sex=2] | 0a | . | . | . | . | . |
|  | ageatscan | -0.0221 | 0.007 | -3.263 | 0.003 | -0.0358 | -0.0083 |
| ****DP_HA*Sex**** | Intercept | 1.000 | 0.036 | 31.174 | <.001 | 1.000 | 1.000 |
|  | DP_HA | 0.0158 | 0.043 | 0.370 | 0.714 | -0.0713 | 0.000 |
|  | [Sex=1] * DP_HA | -0.0823 | 0.063 | -1.301 | 0.203 | 0.000 | 0.0466 |
|  | [Sex=1] | 0.0685 | 0.045 | 1.516 | 0.139 | -0.0235 | 0.000 |
|  | [Sex=2] | 0a | . | . | . | . | . |
|  | ageatscan | -0.0194 | 0.007 | -2.870 | 0.007 | -0.0331 | -0.0056 |
| ****DP_NE*Sex**** | Intercept | 1.000 | 0.036 | 31.302 | <.001 | 1.000 | 1.000 |
|  | DP_NE | 0.0291 | 0.039 | 0.752 | 0.458 | -0.0498 | 0.000 |
|  | [Sex=1] * DP_NE | -0.0961 | 0.062 | -1.540 | 0.133 | 0.000 | 0.0310 |
|  | [Sex=1] | 0.0799 | 0.045 | 1.772 | 0.086 | -0.0120 | 0.000 |
|  | [Sex=2] | 0a | . | . | . | . | . |
|  | ageatscan | -0.0186 | 0.007 | -2.746 | 0.010 | -0.0324 | -0.0048 |
| ****DP_CS*Sex**** | Intercept | 1.000 | 0.048 | 24.293 | <.001 | 1.000 | 1.000 |
|  | DP_CS | -0.0066 | 0.051 | -0.131 | 0.897 | 0.000 | 0.0966 |
|  | [Sex=1] * DP_CS | -0.0436 | 0.081 | -0.538 | 0.594 | 0.000 | 0.000 |
|  | [Sex=1] | 0.0510 | 0.065 | 0.779 | 0.442 | -0.0824 | 0.000 |
|  | [Sex=2] | 0a | . | . | . | . | . |
|  | ageatscan | -0.0203 | 0.007 | -2.995 | 0.005 | -0.0341 | -0.0065 |
| ****Fearbias*Sex**** | Intercept | 1.000 | 0.023 | 49.151 | <.001 | 1.000 | 1.000 |
|  | Fearbias | 0.0020 | 0.040 | 0.049 | 0.961 | -0.0790 | 0.0830 |
|  | [Sex=1] * Fearbias | -0.139 | 0.067 | -2.076 | **0.046** | 0.000 | -0.0026 |
|  | [Sex=1] | 0.0270 | 0.016 | 1.682 | 0.102 | -0.0057 | 0.0596 |
|  | [Sex=2] | 0a | . | . | . | . | . |
|  | ageatscan | -0.0205 | 0.006 | -3.307 | 0.002 | -0.0331 | -0.0079 |

Table 28. Associations between newborn right amygdala mean diffusivity (MD) and disengagement probaabilities (DPs) from fearful (FE), neutral (NE), happy (HA), and control (CS; non-face) pictures, as well as fear bias, at **60** months, with sex as an interaction term.

| Model | Parameter | B | Std. Error | t | P | 95% CI Lower | 95% CI Upper |
| --- | --- | --- | --- | --- | --- | --- | --- |
| DP_FE*****Sex**** | Intercept | 1.128 | 0.042 | 26.813 | <.001 | 1.042 | 1.213 |
|  | DP_FE | -0.010 | 0.047 | -0.211 | 0.834 | -0.107 | 0.087 |
|  | [Sex=1] * DP_FE | 0.050 | 0.070 | 0.711 | 0.482 | -0.093 | 0.193 |
|  | [Sex=2] * DP_FE | 0a | . | . | . | . | . |
|  | [Sex=1] | -0.012 | 0.044 | -0.263 | 0.794 | -0.100 | 0.077 |
|  | [Sex=2] | 0a | . | . | . | . | . |
|  | ageatscan | -0.009 | 0.007 | -1.187 | 0.244 | -0.024 | 0.006 |
| DP_HA*****Sex**** | Intercept | 1.087 | 0.041 | 26.555 | <.001 | 1.004 | 1.170 |
|  | DP_HA | 0.040 | 0.048 | 0.823 | 0.417 | -0.058 | 0.138 |
|  | [Sex=1] * DP_HA | -0.074 | 0.071 | -1.040 | 0.306 | -0.219 | 0.071 |
|  | [Sex=2] * DP_HA | 0a | . | . | . | . | . |
|  | [Sex=1] | 0.067 | 0.051 | 1.325 | 0.195 | -0.036 | 0.171 |
|  | [Sex=2] | 0a | . | . | . | . | . |
|  | ageatscan | -0.007 | 0.008 | -0.899 | 0.375 | -0.022 | 0.009 |
| DP_NE*****Sex**** | Intercept | 1.108 | 0.041 | 27.038 | <.001 | 1.025 | 1.192 |
|  | DP_NE | 0.002 | 0.044 | 0.036 | 0.972 | -0.088 | 0.092 |
|  | [Sex=1] * DP_NE | -0.041 | 0.071 | -0.578 | 0.567 | -0.186 | 0.104 |
|  | [Sex=2] * DP_NE | 0a | . | . | . | . | . |
|  | [Sex=1] | 0.045 | 0.051 | 0.883 | 0.384 | -0.059 | 0.150 |
|  | [Sex=2] | 0a | . | . | . | . | . |
|  | ageatscan | -0.005 | 0.008 | -0.686 | 0.498 | -0.021 | 0.010 |
| DP_CS*****Sex**** | Intercept | 1.106 | 0.053 | 20.837 | <.001 | 0.998 | 1.214 |
|  | DP_CS | 0.009 | 0.057 | 0.153 | 0.879 | -0.107 | 0.124 |
|  | [Sex=1] * DP_CS | -0.038 | 0.091 | -0.416 | 0.680 | -0.222 | 0.147 |
|  | [Sex=2] * DP_CS | 0a | . | . | . | . | . |
|  | [Sex=1] | 0.047 | 0.073 | 0.647 | 0.522 | -0.102 | 0.196 |
|  | [Sex=2] | 0a | . | . | . | . | . |
|  | ageatscan | -0.006 | 0.008 | -0.824 | 0.416 | -0.022 | 0.009 |
| Fearbias*****Sex**** | Intercept | 1.113 | 0.027 | 41.707 | <.001 | 1.058 | 1.167 |
|  | Fearbias | 0.028 | 0.045 | 0.620 | 0.539 | -0.064 | 0.120 |
|  | [Sex=1] * Fearbias | -0.150 | 0.077 | -1.950 | 0.060 | -0.306 | 0.007 |
|  | [Sex=2] * Fearbias | 0a | . | . | . | . | . |
|  | [Sex=1] | 0.032 | 0.018 | 1.726 | 0.094 | -0.006 | 0.069 |
|  | [Sex=2] | 0a | . | . | . | . | . |
|  | ageatscan | -0.007 | 0.007 | -1.017 | 0.317 | -0.022 | 0.007 |

Table 29. Associations between newborn left amygdala mean diffusivity (MD) and emotion- specific bias scores (neutral bias, happy bias, and fearful bias), calculated by subtracting disengagement probabilities (DPs) in each condition from the scrambled-control picture at **60** months.

| Model | Parameter | B | Std. Error | t | P | 95% CI Lower | 95% CI Upper |
| --- | --- | --- | --- | --- | --- | --- | --- |
| ****FE_bias**** | Intercept | 1.000 | 0.02464 | 47.271 | <.001 | 1.000 | 1.000 |
|  | FE_bias | -0.03115 | 0.02885 | -1.080 | 0.288 | -0.08977 | 0.02747 |
|  | ageatscan | -0.02114 | 0.00631 | -3.353 | 0.002 | -0.03395 | -0.00833 |
| ****HA_bias**** | Intercept | 1.000 | 0.02573 | 44.944 | <.001 | 1.000 | 1.000 |
|  | HA_bias | 0.00984 | 0.03091 | 0.318 | 0.752 | -0.05297 | 0.07264 |
|  |  |  |  |  |  |  |  |
| ****NE_bias**** | Intercept | 1.000 | 0.02576 | 45.052 | <.001 | 1.000 | 1.000 |
|  | NE_bias | -0.00534 | 0.03442 | -0.155 | 0.878 | -0.07528 | 0.06460 |
|  | ageatscan | -0.02145 | 0.00655 | -3.274 | 0.002 | -0.03477 | -0.00814 |

Table 30. Associations between newborn right amygdala mean diffusivity (MD) and emotion- specific bias scores (neutral bias, happy bias, and fearful bias), calculated by subtracting disengagement probabilities (DPs) in each condition from the scrambled-control picture at **60** months.

| Model | Parameter | B | Std. Error | t | P | 95% CI Lower | 95% CI Upper |
| --- | --- | --- | --- | --- | --- | --- | --- |
| ****FE_bias**** | Intercept | 1.124 | 0.028 | 40.431 | <.001 | 1.068 | 1.181 |
|  | FE_bias | -0.011 | 0.033 | -0.348 | 0.730 | -0.077 | 0.055 |
|  | ageatscan | -0.007 | 0.007 | -0.938 | 0.355 | -0.021 | 0.008 |
| ****HA_bias**** | Intercept | 1.122 | 0.029 | 39.179 | <.001 | 1.064 | 1.180 |
|  | HA_bias | 0.000 | 0.034 | -0.003 | 0.997 | -0.070 | 0.070 |
|  | ageatscan | -0.007 | 0.007 | -0.920 | 0.364 | -0.022 | 0.008 |
| ****NE_bias**** | Intercept | 1.118 | 0.029 | 39.168 | <.001 | 1.060 | 1.176 |
|  | NE_bias | 0.018 | 0.038 | 0.484 | 0.632 | -0.059 | 0.096 |
|  | ageatscan | -0.006 | 0.007 | -0.824 | 0.416 | -0.021 | 0.009 |

Table 31. Associations between newborn left amygdala mean diffusivity (MD) and emotion- specific bias scores (neutral bias, happy bias, and fearful bias), calculated by subtracting disengagement probabilities (DPs) in each condition from the scrambled-control picture at **60** months with sex as an interaction term.

| Model | Parameter | B | Std. Error | t | P | 95% CI Lower | 95% CI Upper |
| --- | --- | --- | --- | --- | --- | --- | --- |
| ****FE_bias*Sex**** | Intercept | 1.000 | 0.02505 | 46.252 | <.001 | 1.000 | 1.000 |
|  | FE_bias | -0.01934 | 0.03464 | -0.558 | 0.580 | -0.08989 | 0.05121 |
|  | [Sex=1] * FE_bias | -0.05962 | 0.06448 | -0.925 | 0.362 | 0.000 | 0.07172 |
|  | [Sex=1] | 0.03087 | 0.02111 | 1.462 | 0.153 | -0.01213 | 0.07386 |
|  | ageatscan | -0.02204 | 0.00632 | -3.486 | 0.001 | -0.03491 | -0.00916 |
| ****HA_bias*Sex**** | Intercept | 1.000 | 0.02601 | 44.406 | <.001 | 1.000 | 1.000 |
|  | HA_bias | -0.01767 | 0.03747 | -0.472 | 0.640 | -0.09400 | 0.05866 |
|  | [Sex=1] * HA_bias | 0.07417 | 0.07346 | 1.010 | 0.320 | -0.07547 | 0.000 |
|  | [Sex=1] | 0.00391 | 0.01936 | 0.202 | 0.841 | -0.03553 | 0.04335 |
|  | ageatscan | -0.02177 | 0.00666 | -3.271 | 0.003 | -0.03533 | -0.00821 |
| ****NE_bias*Sex**** | Intercept | 1.000 | 0.02594 | 44.578 | <.001 | 1.000 | 1.000 |
|  | NE_bias | -0.03892 | 0.04191 | -0.929 | 0.360 | 0.000 | 0.04644 |
|  | [Sex=1] * NE_bias | 0.08504 | 0.07252 | 1.173 | 0.250 | -0.06268 | 0.000 |
|  | [Sex=1] | 0.00592 | 0.01767 | 0.335 | 0.740 | -0.03007 | 0.04191 |
|  | ageatscan | -0.02166 | 0.00658 | -3.293 | 0.002 | -0.03505 | -0.00826 |

Table 32. Associations between newborn right amygdala mean diffusivity (MD) and emotion- specific bias scores (neutral bias, happy bias, and fearful bias), calculated by subtracting disengagement probabilities (DPs) in each condition from the scrambled-control picture at **60** months with sex as an interaction term.

| Model | Parameter | B | Std. Error | t | P | 95% CI Lower | 95% CI Upper |
| --- | --- | --- | --- | --- | --- | --- | --- |
| ****FE_bias*Sex**** | Intercept | 1.116 | 0.028 | 39.921 | <.001 | 1.060 | 1.173 |
|  | FE_bias | 0.010 | 0.039 | 0.258 | 0.798 | -0.069 | 0.089 |
|  | [Sex=1] * FE_bias | -0.094 | 0.072 | -1.300 | 0.203 | -0.240 | 0.053 |
|  | [Sex=1] | 0.040 | 0.024 | 1.677 | 0.103 | -0.008 | 0.088 |
|  | ageatscan | -0.008 | 0.007 | -1.107 | 0.276 | -0.022 | 0.007 |
| ****HA_bias*Sex**** | Intercept | 1.119 | 0.029 | 38.532 | <.001 | 1.060 | 1.178 |
|  | HA_bias | -0.026 | 0.042 | -0.619 | 0.540 | -0.111 | 0.059 |
|  | [Sex=1] * HA_bias | 0.055 | 0.082 | 0.668 | 0.509 | -0.112 | 0.222 |
|  | [Sex=1] | 0.011 | 0.022 | 0.531 | 0.599 | -0.033 | 0.056 |
|  | ageatscan | -0.008 | 0.007 | -1.070 | 0.292 | -0.023 | 0.007 |
| ****NE_bias*Sex**** | Intercept | 1.114 | 0.029 | 37.947 | <.001 | 1.054 | 1.173 |
|  | NE_bias | 0.004 | 0.047 | 0.078 | 0.939 | -0.093 | 0.100 |
|  | [Sex=1] * NE_bias | 0.025 | 0.082 | 0.309 | 0.760 | -0.142 | 0.192 |
|  | [Sex=1] | 0.013 | 0.020 | 0.669 | 0.508 | -0.027 | 0.054 |
|  | ageatscan | -0.007 | 0.007 | -0.885 | 0.383 | -0.022 | 0.009 |
